# Reproducibility in the absence of selective reporting: An illustration from large‐scale brain asymmetry research

**DOI:** 10.1002/hbm.25154

**Published:** 2020-08-25

**Authors:** Xiang‐Zhen Kong, ENIGMA Laterality Working Group, Xiang‐Zhen Kong, Samuel R. Mathias, Tulio Guadalupe, Christoph Abé, Ingrid Agartz, Theophilus N. Akudjedu, Andre Aleman, Saud Alhusaini, Nicholas B. Allen, David Ames, Ole A. Andreassen, Alejandro Arias Vasquez, Nicola J. Armstrong, Phil Asherson, Felipe Bergo, Mark E. Bastin, Albert Batalla, Jochen Bauer, Bernhard T Baune, Ramona Baur‐Streubel, Joseph Biederman, Sara K. Blaine, Premika Boedhoe, Erlend Bøen, Anushree Bose, Janita Bralten, Daniel Brandeis, Silvia Brem, Henry Brodaty, Dilara Yüksel, Samantha J. Brooks, Jan Buitelaar, Christian Bürger, Robin Bülow, Vince Calhoun, Anna Calvo, Erick Jorge Canales‐Rodríguez, Dara M. Cannon, Elisabeth C. Caparelli, Francisco X. Castellanos, Fernando Cendes, Tiffany Moukbel Chaim‐Avancini, Kaylita Chantiluke, Qun‐lin Chen, Xiayu Chen, Yuqi Cheng, Anastasia Christakou, Vincent P. Clark, David Coghill, Colm G. Connolly, Annette Conzelmann, Aldo Córdova‐Palomera, Janna Cousijn, Tim Crow, Ana Cubillo, Udo Dannlowski, Sara Ambrosino de Bruttopilo, Patrick de Zeeuw, Ian J. Deary, Damion V. Demeter, Adriana Di Martino, Erin W Dickie, Bruno Dietsche, Nhat Trung Doan, Colin P. Doherty, Alysa Doyle, Sarah Durston, Eric Earl, Stefan Ehrlich, Carl Johan Ekman, Torbjørn Elvsåshagen, Jeffery N. Epstein, Damien A. Fair, Stephen V. Faraone, Guillén Fernández, Claas Flint, Geraldo Busatto Filho, Katharina Förster, Jean‐Paul Fouche, John J. Foxe, Thomas Frodl, Paola Fuentes‐Claramonte, Janice M. Fullerton, Hugh Garavan, Danielle do Santos Garcia, Ian H. Gotlib, Anna E. Goudriaan, Hans Jörgen Grabe, Nynke A. Groenewold, Dominik Grotegerd, Oliver Gruber, Tiril Gurholt, Jan Haavik, Tim Hahn, Narelle K. Hansell, Mathew A. Harris, Catharina A. Hartman, Maria del Carmen Valdés Hernández, Dirk Heslenfeld, Robert Hester, Derrek Paul Hibar, Beng‐Choon Ho, Tiffany C. Ho, Pieter J. Hoekstra, Ruth J. van Holst, Martine Hoogman, Marie F. Høvik, Fleur M. Howells, Kenneth Hugdahl, Chaim Huyser, Martin Ingvar, Akari Ishikawa, Anthony James, Neda Jahanshad, Terry L. Jernigan, Erik G Jönsson, Vasily Kaleda, Clare Kelly, Michael Kerich, Matcheri S. Keshavan, Sabin Khadka, Tilo Kircher, Gregor Kohls, Kerstin Konrad, Ozlem Korucuoglu, Bernd Krämer, Axel Krug, Jonna Kuntsi, Jun Soo Kwon, Nanda Lambregts‐Rommelse, Mikael Landén, Luisa Lázaro, Irina Lebedeva, Rhoshel Lenroot, Klaus‐Peter Lesch, Qinqin Li, Kelvin O. Lim, Jia Liu, Christine Lochner, Edythe D. London, Valentina Lorenzetti, Michelle Luciano, Maartje Luijten, Astri J. Lundervold, Scott Mackey, Frank P. MacMaster, Sophie Maingault, Charles B. Malpas, Ulrik F. Malt, David Mataix‐Cols, Rocio Martin‐Santos, Andrew R. Mayer, Hazel McCarthy, Sarah Medland, Mitul Metha, Philip B. Mitchell, Bryon A. Mueller, Susana Muñoz Maniega, Bernard Mazoyer, Colm McDonald, Quinn McLellan, Katie L. McMahon, Genevieve McPhilemy, Reza Momenan, Angelica M. Morales, Janardhanan C. Narayanaswamy, José Carlos Vasques Moreira, Stener Nerland, Liam Nestor, Erik Newman, Joel T. Nigg, Jan Egil Nordvik, Stephanie Novotny, Eileen Oberwelland Weiss, Ruth L. O'Gorman, Jaap Oosterlaan, Bob Oranje, Catherine Orr, Bronwyn Overs, Yannis Paloyelis, Paul Pauli, Martin Paulus, Kerstin Jessica Plessen, Georg G. von Polier, Edith Pomarol‐Clotet, Maria J. Portella, Jiang Qiu, Joaquim Radua, Josep Antoni Ramos‐Quiroga, Y.C. Janardhan Reddy, Andreas Reif, Gloria Roberts, Pedro Rosa, Katya Rubia, Matthew D. Sacchet, Perminder S. Sachdev, Raymond Salvador, Lianne Schmaal, Martin Schulte‐Rüther, Lizanne Schweren, Jochen Seitz, Mauricio Henriques Serpa, Philip Shaw, Elena Shumskaya, Timothy J. Silk, Alan N. Simmons, Egle Simulionyte, Rajita Sinha, Zsuzsika Sjoerds, Runar Elle Smelror, Joan Carlos Soliva, Nadia Solowij, Fabio Luisde Souza‐Duran, Scott R. Sponheim, Dan J. Stein, Elliot A. Stein, Michael Stevens, Lachlan T. Strike, Gustavo Sudre, Jing Sui, Leanne Tamm, Hendrik S. Temmingh, Robert J. Thoma, Alexander Tomyshev, Giulia Tronchin, Jessica Turner, Anne Uhlmann, Theo G.M. van Erp, Odile A. van den Heuvel, Dennis van der Meer, Liza van Eijk, Alasdair Vance, Ilya M. Veer, Dick J. Veltman, Ganesan Venkatasubramanian, Oscar Vilarroya, Yolanda Vives‐Gilabert, Aristotle N Voineskos, Henry Völzke, Daniella Vuletic, Susanne Walitza, Henrik Walter, Esther Walton, Joanna M. Wardlaw, Wei Wen, Lars T. Westlye, Christopher D. Whelan, Tonya White, Reinout W. Wiers, Margaret J. Wright, Katharina Wittfeld, Tony T. Yang, Clarissa L. Yasuda, Yuliya Yoncheva, Murat Yücel, Je‐Yeon Yun, Marcus Vinicius Zanetti, Zonglei Zhen, Xing‐xing Zhu, Georg C. Ziegler, Greig I. de Zubicaray, Marcel Zwiers, Karolinska Schizophrenia Project, David C. Glahn, Fabrice Crivello, Simon E. Fisher, Paul M. Thompson, Clyde Francks, Lars Farde, Lena Flyckt, Göran Engberg, Sophie Erhardt, Helena Fatouros‐Bergman, Simon Cervenka, Lilly Schwieler, Fredrik Piehl, Ingrid Agartz, Karin Collste, Pauliina Victorsson, Anna Malmqvist, Mikael Hedberg, Funda Orhan, Carl Sellgren, Clyde Francks

**Affiliations:** ^1^ Language and Genetics Department Max Planck Institute for Psycholinguistics Nijmegen The Netherlands; ^2^ Department of Psychology and Behavioral Sciences Zhejiang University Hangzhou China; ^3^ Donders Institute for Brain, Cognition and Behaviour Radboud University Nijmegen The Netherlands

**Keywords:** multisite collaboration, *P*‐hacking, publication bias, reproducibility, team science

## Abstract

The problem of poor reproducibility of scientific findings has received much attention over recent years, in a variety of fields including psychology and neuroscience. The problem has been partly attributed to publication bias and unwanted practices such as *p*‐hacking. Low statistical power in individual studies is also understood to be an important factor. In a recent multisite collaborative study, we mapped brain anatomical left–right asymmetries for regional measures of surface area and cortical thickness, in 99 MRI datasets from around the world, for a total of over 17,000 participants. In the present study, we revisited these hemispheric effects from the perspective of reproducibility. Within each dataset, we considered that an effect had been reproduced when it matched the meta‐analytic effect from the 98 other datasets, in terms of effect direction and significance threshold. In this sense, the results within each dataset were viewed as coming from separate studies in an “ideal publishing environment,” that is, free from selective reporting and *p* hacking. We found an average reproducibility rate of 63.2% (*SD* = 22.9%, min = 22.2%, max = 97.0%). As expected, reproducibility was higher for larger effects and in larger datasets. Reproducibility was not obviously related to the age of participants, scanner field strength, *FreeSurfer* software version, cortical regional measurement reliability, or regional size. These findings constitute an empirical illustration of reproducibility in the absence of publication bias or *p* hacking, when assessing realistic biological effects in heterogeneous neuroscience data, and given typically‐used sample sizes.

## Graphical abstract

Region‐wise effect sizes and reproducibility rates of hemispheric asymmetry effects. In general, effects with higher effect sizes showed higher reproducibility, given the same conditions (e.g., sample size and data heterogeneity).

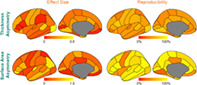

## 1 INTRODUCTION

The issue of reproducibility has received considerable attention in a variety of fields including medicine (Prinz, Schlange, & Asadullah, 2011), psychology (Aarts et al., 2015; R. A. Klein et al., 2014) and neuroscience (Button et al., 2013; Wager, Lindquist, Nichols, Kober, & Van Snellenberg, 2009). Poor reproducibility has been partly attributed to reporting bias and problematic practices such as selective reporting of outcomes (i.e., *p*‐hacking) (Aarts et al., 2015; Baker, 2016; Bakker, van Dijk, & Wicherts, 2012; Ioannidis, 2005, 2008; Ioannidis, Munafo, Fusar‐Poli, Nosek, & David, 2014; John, Loewenstein, & Prelec, 2012; Simmons, Nelson, & Simonsohn, 2011). This situation has resulted in multiple calls for more reproducible research (e.g., Benjamin et al., 2018; Button et al., 2013; O. Klein, Hardwicke, et al., 2018; Poldrack et al., 2017; Valentin Amrhein, 2017). For example, the Open Science Framework has been set up as a free and open source project management resource for researchers across the entire study cycle. In addition, the Transparency and Openness Promotion (TOP) Guidelines (Nosek et al., 2015) have been proposed to improve the quality and credibility of scientific literature. In neuroimaging studies, problems such as flexibility in data analysis have been widely discussed, and best practices have been proposed to ensure that neuroimaging studies can produce meaningful and reliable results (Poldrack et al., 2017). The reproducibility rate was not found to correlate with levels of experience and expertise of study authors, in a replication study of previous findings in psychology (Aarts et al., 2015), which suggests that some practices will not improve merely through training, and that other factors influence reproducibility within the current research convention.

Among these factors, low statistical power is now well understood to contribute to the reproducibility problem (Button et al., 2013; Ioannidis, 2005), although it was only ranked number three behind “*Selective reporting*” and “*Pressure to publish*” in a recent Nature survey (Baker, 2016). The positive predictive value (PPV), that is, the probability that a “positive” research finding reflects a true effect, has been formulated as a function of the prior probability of the effect being real (*R*, the prestudy odds), the statistical power of the study (1 − *β*; *β* is the Type II error), and the level of statistical significance required (*α*; *α* is the Type I error, for example, 0.05 or 0.01): PPV = (1 − *β*)*R*/([1 − *β*]*R* + *α*)) (Button et al., 2013; Ioannidis, 2005). For example, it is evident that a research finding is more likely true than false (i.e., PPV > 50%) if (1 − *β*)*R* > *α*. However, in many cases the true effect size is unknown a priori, and/or the prestudy odds are unknown. This problem is then further complicated by potentially selective reporting or other problematic practices.

The present study aimed to illustrate the reproducibility of human MRI results in an unusual setting where a priori knowledge of the statistical power and prestudy odds was not necessary, and in the absence of selective reporting. This was possible because we leveraged summary statistics from a previous study performed via a worldwide collaborative network known as the Enhancing NeuroImaging Genetics through Meta‐Analysis (ENIGMA) consortium (Thompson et al., 2014). In that study, the ENIGMA consortium mapped left–right hemispheric asymmetry effects on 70 regional and total cortical gray matter metrics, in over 17,000 individuals from 99 datasets (Kong et al., 2018). Hemispheric asymmetry is a key feature of human brain organization, and altered brain asymmetry has been linked to various cognitive and neuropsychiatric disorders (Carrion‐Castillo et al., 2020; Kong, Boedhoe, et al., 2020, Kong, Postema, et al., 2019; Kong et al., 2020; Postema et al., 2019; Toga & Thompson, 2003; Zhen et al., 2017). For the study by Kong et al. (2018), analysis plans and scripts were prepared by a central site and then sent out to many separate sites worldwide to run on their own datasets. Finally, outputs from each dataset were sent back to the central site and combined by meta‐analysis methodology, with no results‐based selection applied to any of the datasets. Thus, we can consider summary statistics from the 99 datasets as being from an “ideal reporting environment”, free from reporting bias, or other potentially problematic practices such as *p*‐hacking.

If we assume the meta‐analytic effect sizes reported by Kong et al. (2018) to represent the “true” hemispheric asymmetry effect sizes, in this way we have access to a real‐world setting for examining reproducibility across 99 datasets in the absence of selective reporting, which can provide a useful illustration of how consistently realistic biological effects in MRI data can be detected when surveying cohorts worldwide. If we wanted to address this question with actual papers in the literature, then an ideal publishing environment would first need to be established, free from selective reporting. This seems impossible in the current era, because many journals and scientists are incentivized to report statistically significant results, while leaving nonsignificant findings unpublished (known as the *file‐drawer* effect).

Specifically, Kong et al. (2018) analyzed thickness and area measures for each of 34 brain regions based on the Desikan‐Killiany atlas from *FreeSurfer*, as well as entire hemisphere‐level average thickness and total area (Fischl, 2012), for a total of 70 left–right hemispheric effects. For the present study of reproducibility, we considered each of these 70 asymmetry effects as a single research question, for example, does the parahippocampal gyrus show left–right asymmetrical thickness, on average, in the human brain? We further considered that each of these questions had been asked 99 times in separate datasets, with a range of different sample sizes, scanning equipment and parameters (although image processing with *Freesurfer* was harmonized across datasets). Our goal was to use these data to assess reproducibility of hemispheric asymmetry effects not only in an “ideal publishing environment”, but also in the context of extensive dataset heterogeneity, which is a feature of the real‐world literature.

## 2 MATERIALS AND METHODS

### 2.1 Datasets

We used publicly available summary statistics from the previously published ENIGMA cortical asymmetry project (http://conxz.net/neurohemi/) (Kong et al., 2018). That study used data from 17,141 healthy participants from 99 separate datasets, each of which showed different age distributions, and were from diverse ethnic backgrounds (Table S1). Participants were drawn from the general population or were healthy controls from clinical case–control studies (affected individuals were not included). In most cases each participating site contributed one dataset, but there were seven sites that contributed more than one dataset from distinct studies (Table S1), for example when different scanners were used (1.5T and 3T: e.g., OCD_Cheng_1.5T and OCD_Cheng_3T; OCD_VUmc 1.5T and OCD_VUmc 3T), or different age groups were recruited (such as children and older adults: e.g., NSIOCDS_3T_Adults and NSIOCDS_3T_Child; GBB_ GRADUAL and GBB_OLDERS).

In the present study we analyzed the reproducibility of hemispheric asymmetry effects on paired left–right measures of cortical thickness and surface area, for 34 brain regions based on the Desikan‐Killiany atlas from *FreeSurfer* (Fischl, 2012), as well as entire hemisphere‐level average thickness and total surface area. See Kong et al. (2018) for details about the neuroimaging processing and quality control. Briefly, images were acquired using scanners of different field strengths (1.5T and 3T; Table S1) and all images were analyzed using the automated and validated pipeline “recon‐all” implemented in *FreeSurfer* (Fischl, 2012), although different software versions were possible (version 5.0, 5.1, and 5.3) (Table S1).

Table S1 gives summary information for each dataset: Briefly, the sample size varied across datasets from 14 to 2,326 (median 72); *FreeSurfer* version 5.3 was used exclusively in 91 datasets, version 5.1 exclusively in 6 datasets, version 5.0 in one dataset, and a mixture of 5.1 and 5.3 in one dataset; 63 datasets used a 3T scanner and 29 datasets used a 1.5T scanner; the minimum age across all datasets was 3 years, the maximum age was 90 years. All local institutional review boards permitted the use of extracted measures from the anonymized data.

For each dataset and paired left–right measure, Kong et al. (2018) used paired *t*‐tests to assess inter‐hemispheric differences, and Cohen's *d* was calculated based on each paired *t*‐test, to estimate the hemispheric effect size (i.e., the standardized difference between the mean left and right measures, for a given region and dataset). In the procedure, analysis plans and scripts were prepared by a central site and sent out to each dataset's own site for running the analysis, and finally all outputs for every dataset were sent back to the central site for meta‐analysis. (A laterality index was also used when investigating how factors such as age, sex, and handedness affected brain asymmetry [Kong et al., 2018], but this is not relevant to the present study.)

In addition to the per‐dataset summary statistics and meta‐analysis results from Kong et al. (2018), two additional datasets with MRI data were used for the present study. These were the Human Connectome Project (HCP; https://www.humanconnectome.org/) and Brain Imaging Genetics (BIG; http://cognomics.nl/) datasets. The HCP comprises 1,113 individuals (age 22–37) scanned using the HCP's custom 3T Siemens Skyra, with data processing as previously described (Glasser et al., 2013). For each of the 34 regions in the Desikan‐Killiany atlas, cortical thickness and surface area were derived based on individual T1‐weighted MRI images (HCP pipeline based on *FreeSurfer* version 5.3). The HCP data were only used to define a population mean surface area for each cortical region, which was used to test whether regional size is related to reproducibility of asymmetry effects (see below).

In the BIG dataset, we included T1‐weighted MRI scans of 423 participants (age 18–74) who were scanned twice on separate occasions. Their second scan was done from zero to 2,650 days after their first scan (median = 149; mean = 325, *SD* = 432). From these data we could calculate the reliability of regional measures, and then test for a relation of reliability to reproducibility (see below). BIG data were scanned using either a 1.5T Siemens Avanto or Sonata scanner, or a 3.0T Siemens TIM Trio or Skyra scanner (see Table S2 for the numbers of participants by scanner). *FreeSurfer* version 5.3 was used for deriving cortical thickness and surface area for each of the 34 regions in the Desikan‐Killiany atlas.

### 2.2 Estimation of the “true” effects

Given a lack of consistency of brain asymmetry findings in earlier literature, Kong et al. (2018) performed the largest ever study of this issue, based on at least an order of magnitude more participants than any previous study. For each regional or total thickness or surface area measure, a paired *t*‐test (i.e., paired within subject) was used to compare the mean left and right measure, separately within each dataset. These *t*‐tests provided the hemispheric effects, and the outputs from each of the 99 datasets were combined using inverse variance‐weighted random‐effect meta‐analysis, with the R package *metafor*, version 1.9‐9. This method tests one overall effect, while weighting each dataset's contribution by the inverse of its corresponding sampling variance. Thus, unlike fixed‐effect meta‐analysis, this method takes into account variability across difference studies. In addition, test statistics in the meta‐analyses were computed based on a standard normal distribution (test = “z” by default). For more details, see (Kong et al., 2018).

A Cohen's d effect size estimate of the population‐level asymmetry was obtained for each hemispheric effect, for each paired left–right measure, that is, the standardized difference between the mean left and right measures. The Cohen's d hemispheric effects derived from the meta‐analytic approach over 99 datasets can be taken as “true” effects representing left–right differences in the average human brain, as measured through this image processing and analysis pipeline. Sixty‐three of the 70 hemispheric effects were significant at *p* ≤ .05 (uncorrected for multiple testing across regions) in the meta‐analysis over 99 datasets, while seven of the effects were not significantly different from zero (*p* > .05) (Kong et al., 2018). We also applied a more stringent significance threshold of 0.05/70 to identify “true” effects surviving multiple testing correction, which resulted in 56 significant effects and 14 nonsignificant effects.

Note that a meta‐analytic approach was taken by Kong et al. (2018) because the central analysis team did not have individual‐level data from most of the 99 datasets. The different sites ran the same script on their data and returned summary statistics to the central team for meta‐analysis. Individual‐level data sharing can require material transfer agreements and additional IRB approvals, so that a meta‐analytic approach was the only way to achieve a timely study of this many datasets. Nonetheless, meta‐analysis and individual‐level‐analysis were found to result in similar effect sizes in a recent, empirical comparison using large‐scale multisite neuroimaging data (Boedhoe et al., 2018).

### 2.3 Estimation of reproducibility

For the present study, the reproducibility rate for a given effect was calculated as the proportion of datasets in which that effect was reproduced. Specifically, we applied a leave‐one‐out strategy for estimation of reproducibility: each effect within each dataset was compared in turn to the corresponding meta‐analytic effect from the 98 other datasets, to avoid sample overlap.

For an effect that was significant at *p* ≤ .05 in a given meta‐analysis of 98 datasets (or more stringently at *p* ≤ .05/70 to correct for multiple comparisons across 70 effects), the effect in the remaining single dataset would need to be in the same left–right direction as in the meta‐analysis, and also be nominally significant within the single dataset (*p* ≤ .05), in order to be counted as reproduced in that dataset.

For an effect that was nonsignificant in the meta‐analysis (*p* > .05, or *p* > .05/70 to correct for multiple comparisons across 70 effects), then the effect would also need to be nonsignificant (*p* > .05) in the single dataset, in order to be counted as reproduced in that dataset. Thus reproducibility in our formulation is not simply a measure of the detection rate of significant effects (i.e., statistical power), but also incorporates the consistency of finding nonsignificant effects in the individual datasets, when those effects were not significant in meta‐analysis.

### 2.4 Reproducibility, effect size, and sample size

**FIGURE 1:**
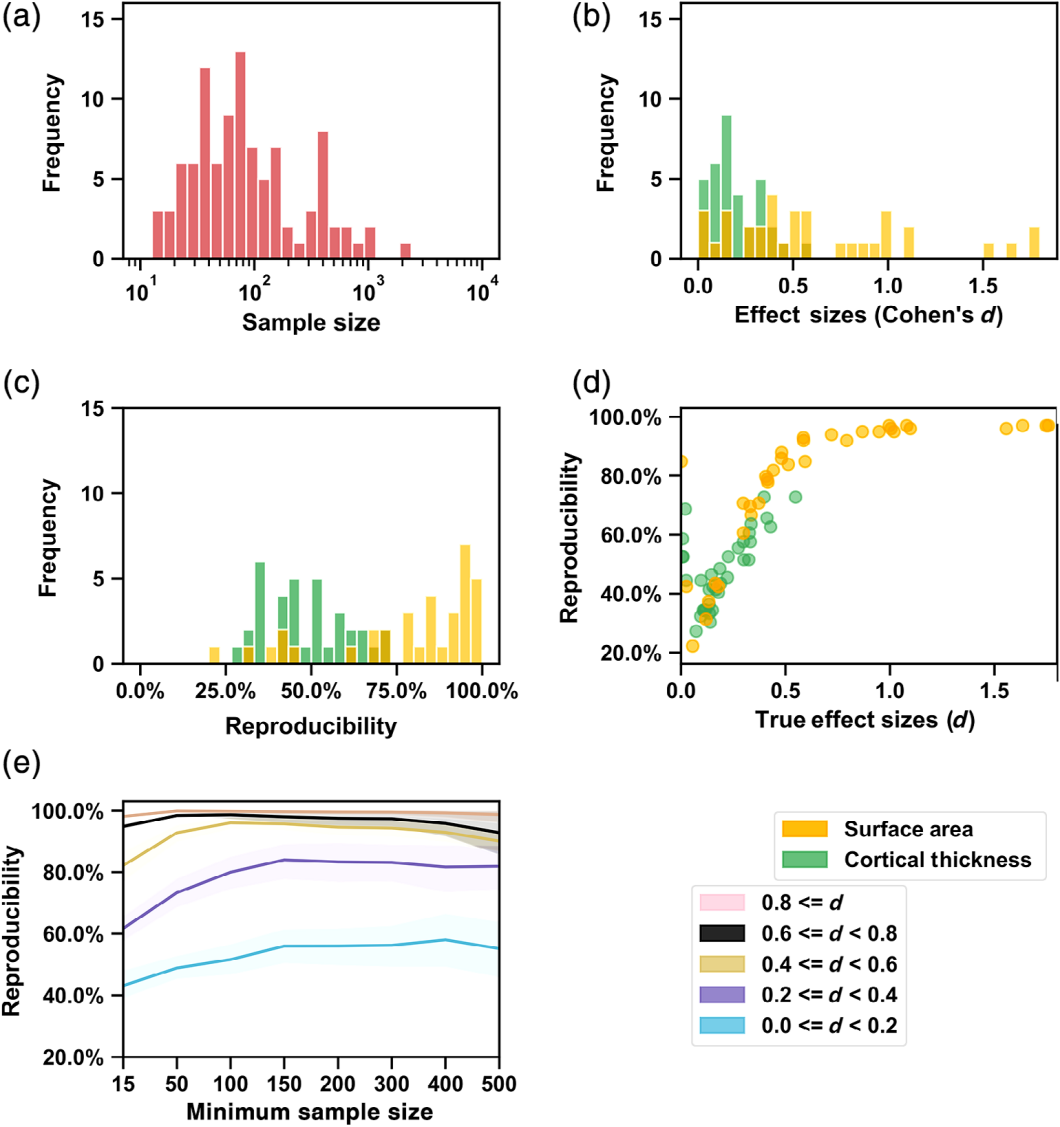
Reproducibility in the absence of selective reporting, estimated based on outputs of the ENIGMA cortical asymmetry project. (a) Sample size distribution of the 99 datasets. (b) Effect size distribution of the 70 hemispheric effects of interest. (c) Reproducibility distribution of the 70 hemispheric effects. The reproducibility was assessed by comparing each dataset in turn to the meta‐analytic effect from the 98 others, to avoid overlap (see Methods). (d) Scatter plot of the correlation between the reproducibility and the effect size. (e) Relations of reproducibility, effect size and dataset sample size. Each line plots the mean and 95% confidence interval for reproducibility. We used the meta‐analytic effect size over all 99 datasets for visualization purposes. The figure key shows the types of cortical measure (orange indicates surface area; green indicates cortical thickness), as well as groupings by true effect sizes

The hemispheric effect size varied across different brain structural measures (Kong et al., 2018), from Cohen's *d* = 0.0015–1.76 (median 0.30) (unsigned magnitudes) (Figure 1). In addition, the sample size varied across datasets, from 14 to 2,326 (median 72) (Figure 1a; Table S1). These variabilities allowed us to illustrate the expected relationships of reproducibility, effect size and sample size. As surface area asymmetries are generally more substantial than cortical thickness asymmetries (Figure 1b) (Kong et al., 2018), we first compared reproducibility rates between the hemispheric effects for these two separate types of measure. We then calculated the Spearman correlation between the “true” effect size and the reproducibility rate across all 70 hemispheric effects, as well as across cortical thickness and surface area hemispheric effects separately.

We also used a descriptive approach to show the relationship between reproducibility and sample size. Specifically, to explore the contribution of sample size of datasets to reproducibility, we calculated the reproducibility rate for each effect using a range of minimum sample size thresholds of 15, 50, 100, 150, 200, 300, 400, and 500. That is, in each case, the reproducibility rate was calculated based on only the datasets with sample sizes above that threshold. This resulted in different subgroups of datasets with varied minimum sample sizes: minimum size 15 as in the main analysis described above (97 datasets in total), and then minimum size 50 (63 datasets), minimum size 100 (37 datasets), minimum size 150 (25 datasets), minimum size 200 (20 datasets), minimum size 300 (19 datasets), minimum size 400 (12 datasets), and minimum size 500 (7 datasets).

In addition, we used a descriptive approach to group the 70 effects based on their meta‐analytic effect sizes (based on all 99 datasets): 0.0 ≤ *d* < 0.2, 0.2 ≤ *d* < 0.4, 0.4 ≤ *d* < 0.6, 0.6 ≤ *d* < 0.8, 0.8 ≤ *d <* 1.8, and summarized the reproducibility rate for each effect size subgroup separately.

The R package *pwr* was used for calculating statistical power to detect meta‐analytic effect sizes in relation to dataset sample sizes.

### 2.5 Reproducibility and data heterogeneity

The majority of the datasets (91 of 99) were processed using *FreeSurfer* version 5.3. Therefore the meta‐analytic effect sizes may particularly reflect this software version, to the extent that different versions of *FreeSurfer* can yield slightly different measures (Gronenschild et al., 2012). We compared the reproducibility rates of the 70 effects (again using the 1 dataset vs. 98 approach) between the datasets using version 5.3 (91 datasets) versus those using version 5.1 (6 datasets), using both the *t*‐test and the nonparametric Mann–Whitney test. (Note that the small number of datasets using version 5.1 limited our ability to test whether *FreeSurfer* version affects reproducibility.) In the same way, we also compared the reproducibility rates of the 70 effects between the 63 datasets with 3T scanning versus the 29 datasets with 1.5T scanning, and between the 18 datasets with maximum age 18 years versus the 36 datasets with minimum age 19 years. We also ran meta‐analyses for the subgroups separately (i.e., 3T/1.5T datasets, and child/adult datasets) to estimate subgroup‐specific “true” effects for calculating reproducibility.

In addition, we investigated the potential relationships between cortical regional variation in reproducibility and two regional properties: regional size (surface area) and measurement reliability, as a proxy of image quality and region‐specific performance of *FreeSurfer*. In terms of variation in region size, the HCP dataset (see above) was used to estimate the population averaged surface area of homologous pairs of left–right regions (i.e., the population mean [*L* + *R*]/2 per region). Spearman correlation across the 34 cortical regions was then used to relate this regional size variable to reproducibility in the ENIGMA results, separately for thickness and area asymmetry effects. In terms of regional measurement reliability (as a proxy for variation in regional image quality and region‐specific performance of *FreeSurfer*), we calculated the intraclass correlation coefficients (ICC) for cortical thickness and surface area measures of each of the 34 regions using the test–retest dataset of 423 participants from the BIG dataset (see above), and then examined the correlations between ICCs and reproducibility rates across the 34 regions (separately for thickness and surface area asymmetry effects).

### 2.6 Data and code sharing

Data used in this study were published summary statistics from the ENIGMA cortical asymmetry project (Kong et al., 2018). Data and scripts for all analyses are available in GitHub (https://github.com/Conxz/illusReproducibility). Additional data were from the Human Connectome Project (https://www.humanconnectome.org/), and the BIG dataset (http://cognomics.nl/).

## 3 RESULTS

### 3.1 Estimating reproducibility

There was an overall mean reproducibility rate of 63.2%, that is, on average 63.2% of the single‐dataset results were consistent with the meta‐analytic true effects (see Methods). A large variability of reproducibility was observed across effects (*SD* = 22.9%, range from 22.2 to 97.0%) (Figure 1c). When using a more stringent significance threshold of 0.05/70 for correcting multiple testing in the meta‐analytic results, the reproducibility remained similar (mean = 64.4%, *SD* = 21.9%), which reflects that the large majority of effects were significant regardless of this correction (63 significant before correction, 56 significant after correction).

**FIGURE 2:**
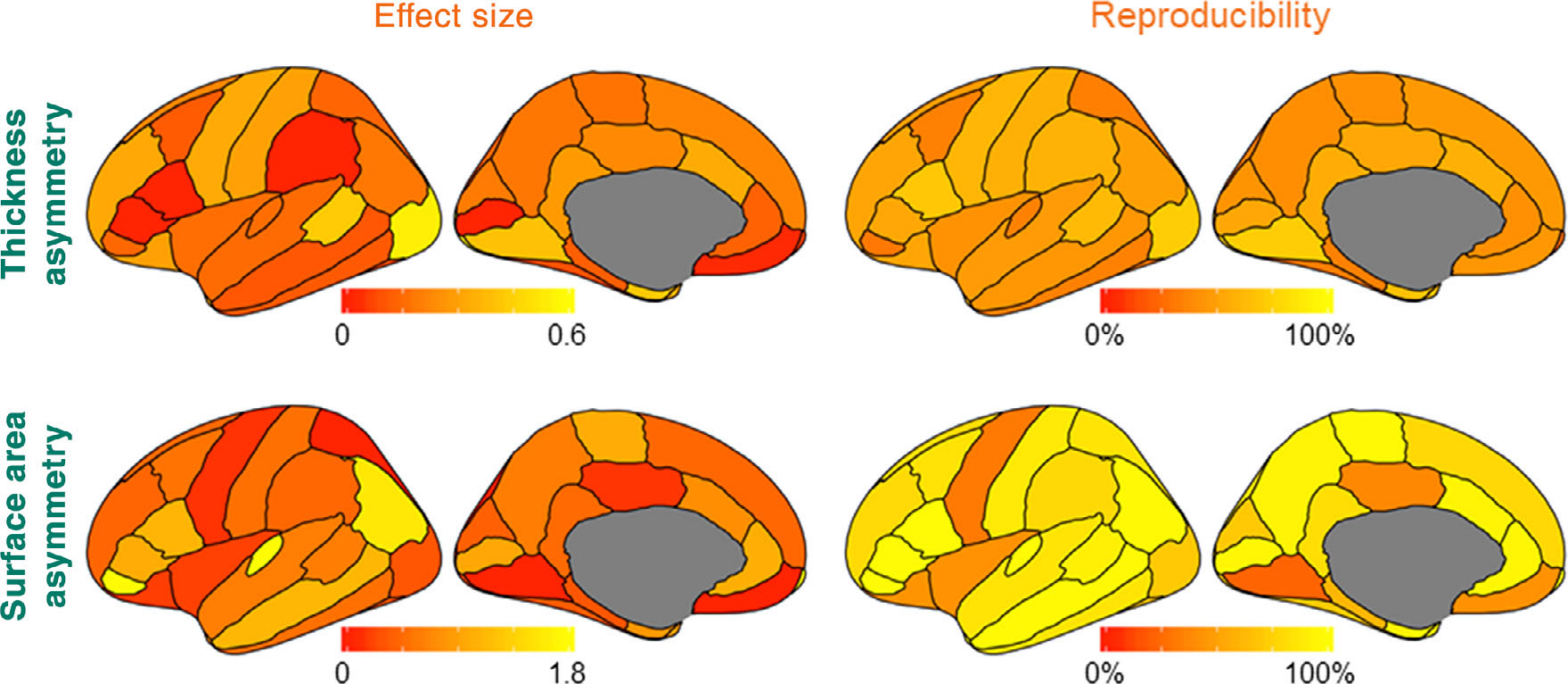
Region‐wise effect sizes and reproducibility rates of hemispheric asymmetry effects. We used the meta‐analytic effect sizes over all 99 datasets for visualization purposes. Effect sizes are in Cohen's *d*

For the whole hemisphere asymmetry effects (i.e., derived from the average cortical thickness over each entire hemisphere, and the total surface area of each hemisphere), the reproducibility rates across datasets were 36.4 and 66.7%, respectively. For regionally specific hemispheric effects, the reproducibility rate across datasets ranged from 27.3 to 72.7% (mean = 48.7%, *SD* = 12.3%), and from 22.2 to 97.0% (mean = 78.4%, *SD* = 21.7%) for cortical thickness and surface area measures, respectively (Figure 2). These findings show that reproducibility is far from perfect, even without any publication bias or potentially problematic practices such as *p*‐hacking.

The overall reproducibility remained similar (62.7%, *SD* = 23.3%) after excluding the two largest individual datasets (i.e., BIG and QTIM), which might have had a disproportionate influence.

### 3.2 Reproducibility, effect size, and sample size

As expected, regionally specific surface area measures (Figure 1b) showed significantly higher reproducibility rates for hemispheric effects than cortical thickness measures (Area vs. Thickness: *t*[33] = 6.84, *p* = 3.11e−09; Mann–Whitney *U* = 985.0, *p* = 6.08e−07) (Figure 1c), as hemispheric effects on surface area are generally larger (Figure 2). As noted above, there were 63 significant effects and 7 nonsignificant effects with an uncorrected significance threshold of 0.05, in the meta‐analysis of 99 datasets. The reproducibility rate of the significant effects ranged from 22.2 to 97% (mean = 63.8%, *SD* = 23.7%), while the reproducibility rate of the nonsignificant effects ranged from 42.4 to 84.8% (mean = 57.7%, *SD* = 13.7%).

Reproducibility showed a significant correlation with the “true” effect size for both types of measure (all measures together, *rho* = .84, *p* = 2.15e−19; thickness, *rho* = .52, *p* = .0017; area, *rho* = .94, *p* = 4.28e−16) (Figure 1d). After excluding the 7 nonsignificant effects, the correlation between reproducibility and effect size became more significant (all measures together, *rho* = .97, *p* = 5.31e−40; thickness, *rho* = .91, *p* = 7.94e−12; area, *rho* = .98, *p* = 3.72e−21). Note that some meta‐analytic effects very close to zero could show relatively high reproducibility (Figure 1d), since in most individual datasets they were also found to be low and nonsignificant as in the meta‐analysis (e.g., surface area asymmetry in superior parietal cortex, *d* = 0.002, reproducibility rate = 84.8%, and cortical thickness asymmetry in the pars opercularis, *d* = 0.02, reproducibility rate = 68.7%) (Figure 2). Other nonsignificant meta‐analytic effects showed relatively lower reproducibility (Figure 1d), which may reflect uncontrolled sources of dataset heterogeneity affecting regional measurement (see below and Discussion).

With a corrected significance threshold of 0.05/70 for identifying the “true” effects, there were 56 significant effects, and 14 nonsignificant effects. In this case, the reproducibility rate for significant effects ranged from 30.3 to 97%, mean = 67.6%, *SD* = 22.3%, and for nonsignificant effects from 25.3 to 84.8%, mean = 52.0%, *SD* = 14.4%.

When examining subgroups of effects and datasets according to thresholds on effect size and sample size, we found that the reproducibility rate increased with the minimum sample size threshold, for each specific range of effect size (Figure 1e). For example, for effects of *d* ≥ 0.6, the reproducibility rate was higher than 90% even when including the datasets with sample sizes as low as 15, while for effects of 0.4 ≤ *d* < 0.6, a minimum sample size of 50 was needed to obtain a reproducibility rate of 90%. Moreover, for effects of 0.2 ≤ *d* < 0.4, a minimum sample size threshold of 100 started to make a reproducibility rate of 80% achievable. In addition, the empirical findings showed that it was impossible to obtain 70% reproducibility for small effects of *d* < 0.2, even with a relatively large minimum sample size threshold of 500.

We also examined the distributions of per‐dataset effect sizes in relation to dataset sample sizes, and the expected power function to detect each meta‐analytic effect (Figure S1). There was considerable variation across datasets around the meta‐analytic effect sizes, and as expected, this variation generally decreased as the sample size increased (Figure S1). However, some of the larger datasets could also yield effects that were discrepant with the corresponding meta‐analytic effects, which again may relate to uncontrolled heterogeneity affecting these datasets (see below and Discussion).

### 3.3 Reproducibility and data heterogeneity

For subgroups of datasets processed with different versions of *FreeSurfer* (version 5.3 in 91 datasets and version 5.1 in 6 datasets) there was no significant difference in reproducibility of the 70 effects: mean reproducibility rate 62.8% (*SD* = 22.8%) for version 5.3 and 64.3% (*SD* = 32.8%) for version 5.1 (*t* = 0.31, *p* = 0.76; Mann–Whitney *U* = 2,693.0, *p* = .31). Similarly, no significant difference was found between the datasets with scanner field strengths of 3T (63 datasets) versus 1.5T (29 datasets): mean reproducibility rate 59.6% (*SD* = 23.3%) for 3T datasets and 66.8% (*SD* = 25.6%) for 1.5T datasets (*t* = 1.73, *p* = .09; Mann–Whitney *U* = 2,920.0, *p* = .503). Furthermore, there was no significant difference in the reproducibility rate of the 70 effects between the 18 datasets with maximum age 18 years (mean reproducibility 59.7%, *SD* = 24.7%) versus the 36 datasets with minimum age 19 years (mean reproducibility 61.9%, *SD* = 24.6%) (*t* = −0.53, *p* = .60, Mann–Whitney *U* = 2,294.5, *p* = .52).

In addition, there were no significant differences of sample size between the subgroups of datasets (version 5.3 vs. 5.1, *t* = *−*0.29, *p* = .77; Mann–Whitney *U* = 203, *p* = .30:3T vs. 1.3T, *t* = −0.78, *p* = .44; Mann–Whitney *U* = 733.0, *p* = .13: Childhood/adult, *t* = −1.41, *p* = .17; Mann–Whitney *U* = 300.5, *p* = .67), so that these factors were not obviously confounded with dataset sample size.

We further ran meta‐analyses for the subgroups separately (i.e., 3T/1.5T datasets, and child/adult datasets) to estimate subgroup‐specific “true” effects for calculating reproducibility, in case the reduced heterogeneity within subsets might boost reproducibility, although this inevitably resulted in lower numbers of datasets within subgroups, compared to the main analysis. Reproducibility of these subgroup‐specific meta‐analysis effects within the subgroups remained similar to the main analysis (3T: 60.6%, *SD* = 23.0%; 1.5T: 69.1%, *SD* = 23.0%; Child: 61.7%, *SD* = 23.3%; Adult: 63.8%, *SD* = 23.0%).

**FIGURE 3:**
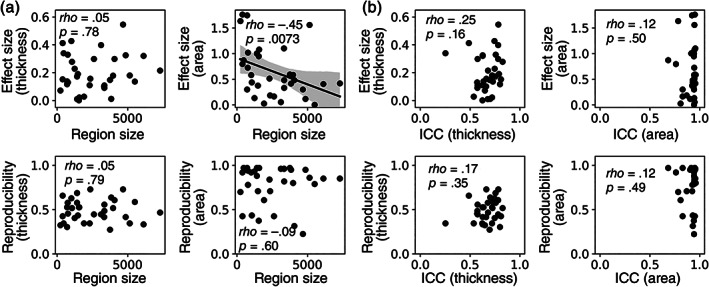
Scatter plots for the relationships between regional sizes, hemispheric asymmetry effect sizes, and reproducibility rates of the asymmetries. (a) Plots for region sizes. (b) Plots for measurement reliability (intraclass correlation coefficient, ICC). Effect sizes are in Cohen's *d*. Unit: thickness = mm, area = mm^2^, region size = mm^2^

Cortical regional size was significantly correlated across the 34 regions with the effect size of surface area asymmetries (*rho* = .45, *p* = .0073), but not with the effect size of thickness asymmetries, and not with the reproducibility rates for either thickness or area asymmetry effects (*p*s > .15; Figure 3a). In terms of measurement reliability (as a proxy for variation in regional image quality and region‐specific performance of *FreeSurfer*) based on 423 twice‐scanned participants, there were no significant correlations between regional measure ICCs and either asymmetry effect sizes or reproducibility rates across the 34 regions (*p*s > .15; Figure 3b). One may ask whether it is useful to apply regional reliability measures from one study to another. We investigated the correlations between our reliability measures and those reported in a previous study using an independent scan‐rescan dataset (Iscan et al., 2015). There were high correlations between the two studies for both thickness (*r* = .75, *p* < .0005) and area measures (*r* = .92, *p* < .0005). These high correlations indicate stable inter‐regional differences of measurement reliability across studies.

Taken together, our results indicate that the potential heterogeneity factors of *FreeSurfer* version, scanner field strength, participant age range in childhood versus adulthood, cortical region size, and regional measurement reliability contribute little to differences in the reproducibility of asymmetry effects, for either cortical thickness or surface area.

## 4 DISCUSSION

In this study, we revisited the summary statistics from a worldwide collaborative neuroscience project that mapped cerebral cortical asymmetry (Kong et al., 2018), to illustrate the reproducibility of realistic biological effects in the absence of *p* hacking or publishing bias, based on heterogeneous neuroscience data and typically‐used sample sizes. Overall, reproducibility was limited, with a mean rate across all effects = 63.2%, lowest reproducibility rate = 22.2%. As expected, sample size and effect size were the primary drivers of reproducibility, while perhaps surprisingly, heterogeneity factors were of limited influence.

Among various factors, low statistical power is now well understood to contribute to the reproducibility problem (Button et al., 2013; Ioannidis, 2005). However, low power was only ranked number three behind “*Selective reporting*” and “*Pressure to publish*” in a recent Nature survey on contributing factors to irreproducible research (Baker, 2016). In an idealized situation, where there is no selective reporting or pressure to publish, we found that the reproducibility was still limited. As expected, the reproducibility rate increased with the true effect size, as well as the sample size of datasets, which together contribute to statistical power. Clearly, to avoid poor reproducibility, a relatively larger sample size is necessary than was available within many of the individual datasets of this study. For example, to obtain a reproducibility rate of 80% for a true effect size of around *d* = 0.4, the sample sizes of individual datasets needed be larger than 100, that is, greater than the median sample size in this study of 99 datasets. There is therefore substantial room to improve reproducibility by increasing sample sizes, even when using currently available methods. Note that the analysis of brain asymmetry involves an inherently paired sample design (i.e., paired left and right measures within subjects), but that the overall picture and principles illustrated here are broadly applicable.

Button et al. (2013) showed that the average statistical power of studies in the neurosciences is low (i.e., around 21%), which is expected to cause low reproducibility, both through false positive and false negative findings. For example, many fMRI studies have traditionally been performed using 10–20 participants (Desmond & Glover, 2002). Our observation that reproducibility is strongly influenced by sample size was expected, and in line with the PPV calculation (Button et al., 2013) mentioned in the Introduction. However, here we have demonstrated this empirically in a situation where a priori knowledge of the statistical power (1 − *β*) and the prestudy odds (*R*) was not necessary, and in a real‐world setting, that is, with similar heterogeneity to the field in general, as regards factors such as scanner field strengths, software versions, demographic differences, and regional differences in measurement reliability. Despite all of this heterogeneity, the primary driver of reproducibility remained the sample size in relation to the effect size, which is an important take‐home message for the field. For example, if the expected effect size (i.e., Cohen's *d*) in a paired‐measure MRI study is below 0.2, then studies with 500 subjects are still not expected to achieve a reproducibility rate of 80%. Consistent with this, a recent study performed an empirical examination of the replicability of “structural brain behavior” associations using a permutation‐based approach (again without any of problems of selective reporting), and concluded that it is relatively unlikely to find an association between behavioral traits and brain morphology with a sample size of less than 500 (replication effect sizes were up to 0.4 [Pearson's *r*]) (Kharabian Masouleh, Eickhoff, Hoffstaedter, Genon, & Alzheimer's Disease Neuroimaging, 2019).

A reproducibility rate of 36% was reported by the Open Science Framework for 100 findings from psychological studies (Aarts et al., 2015), and a reproducibility rate of 54% for 28 classic findings in psychological science was reported by a more recent Many Lab project (R. Klein, Vianello, et al., 2018). Such poor reproducibility has been partly attributed to reporting bias and potentially problematic practices such as selective reporting of outcomes (Aarts et al., 2015; Baker, 2016; Bakker et al., 2012; Ioannidis, 2005, 2008; Ioannidis et al., 2014; John et al., 2012; Simmons et al., 2011). While we do not dispute the likely relevance of these factors, it is interesting to note that the mean reproducibility rate in the present study, where no such factors were in play, was only 63.2%. As the true effect sizes in the present study ranged from zero to large (Cohen's *d* up to 1.8), in this respect they can be taken as broadly comparable to those in the human neuroscience and psychology literature, although the effect size distribution within this range might not be representative of the literature at large.

Varying demographic composition of datasets is another factor likely to influence the reproducibility of findings in human neuroscience, even for such fundamental processes as age‐related change in neural structure (LeWinn, Sheridan, Keyes, Hamilton, & McLaughlin, 2017). However, one study that investigated variation in replicability suggested that the contribution of sample heterogeneity can also be modest (R. Klein, Vianello, et al., 2018). The datasets of the current study differed widely in their age ranges and distributions (Table S1). However, we found no significant difference of reproducibility rates between childhood and adult datasets (thresholded at age 18 years), which suggests that this factor was of limited importance. In terms of technical heterogeneity too, there was variation between datasets in terms of scanner field strength and *FreeSurfer* software version (see Table S1), but again we found that these factors did not significantly relate to reproducibility rates. One reason could be that these factors primarily affect bilateral measures of cortical thickness and surface area, that is, mostly equally for the two hemispheres, such that hemispheric asymmetry effects are relatively robust to these factors. Thus the focus on brain asymmetry may have obscured factors affecting both hemispheres equally. Note that our ability to detect a difference in reproducibility between different versions of *FreeSurfer* was limited, as 91 out of 99 datasets had used version 5.3.

We acknowledge that there remains extensive heterogeneity in other factors such as scanner models and acquisition parameters, which we were not able to consider in this study because variation was too fragmented across the 99 datasets to do meaningful analysis of how they affected reproducibility. Recruitment criteria and strategies were heterogeneous across datasets too, for example whether subjects were selected as healthy controls for disorder case–control studies, or recruited in the context of unselected population studies (we did not use data from case participants from case–control datasets for the present study). Given this heterogeneity, some “nonreplication” of effects could be quite appropriate in certain datasets that comprise specific subgroups or methodological variants, in which particular effects might be of less relevance. Even some of the relatively larger individual datasets could sometimes show markedly different effects from the meta‐analysis (Figure S1). This was likely caused by uncontrolled heterogeneity factors affecting those specific datasets.

Although heterogeneity may have contributed to the overall 63.2% reproducibility rate in this study, we regard it as a strength rather than limitation, as we wished the meta‐analytic effect sizes and reproducibility rates to be valid in the context of the heterogeneity typical of the field. Heterogeneity was not therefore an overarching problem for the present study, but rather fitted its purpose. Nonetheless, future studies may examine how different aspects of MRI dataset heterogeneity influence reproducibility, to gain further insights into the problem that the neuroscience community is facing. It is important to note that, while conceptually related to heterogeneity, the reproducibility rate is also influenced by sample and effect sizes (as discussed above), and as such provides a useful way of examining the replicability of effects under real world conditions.

Neuroimaging studies can involve considerable flexibility regarding data processing and statistical analysis, while inconsistent strategies can also contribute to poor reproducibility and contrasting conclusions (Botvinik‐Nezer et al., 2020; Pauli et al., 2016). For the present study, the pipeline for MRI quality control, processing and analysis was harmonized, so that the impact of this aspect was necessarily limited. Therefore our reproducibility rates may be somewhat idealized, considering how the field typically operates, that is, with different researchers asking similar questions, but in different datasets and using different strategies. In other words, the reproducibility would likely be worse when the processing pipelines and analysis strategies are different.

Another important aspect affecting reproducibility in the literature may be failure to use blinded designs in primary studies, for example so that researchers know the case–control status of participants while processing their data, and inadvertently introduce bias. This is less likely to be relevant for studies based on automated processing of human brain MRI data, unless there would be bias during visual quality control. As regards our study specifically, the visual quality control of *FreeSurfer* segmentations and parcellations was not done with respect to eventual asymmetry measures, and we have no reason to imagine that the inspection of left and right‐hemisphere images was approached differently, on average.

The lowest reproducibility was 22.2%, for a small but significant meta‐analytic effect of *d* = 0.052 (cortical area asymmetry in the lingual gyrus). As discussed above, such low reproducibility is likely due to limited power to detect such small effects, in many of the datasets. There were also seven nonsignificant meta‐analytic effects, with best estimate effect sizes very close to zero, which were considered to have been reproduced when a given dataset also showed no significant effect. With a significance threshold of 0.05/70 for identifying “true” effects in the context of multiple testing of 70 cortical measures, there were 56 significant effects, and 14 nonsignificant effects. Some of the nonsignificant meta‐analytic effects with the lowest effect sizes showed high reproducibility, as nonsignificant effects. However, as the reproducibility rates of some nonsignificant meta‐analytic effects were lower than expected at the alpha level 0.05 (i.e., significant asymmetries were measured in some individual datasets even when the meta‐analysis effect was close to zero and nonsignificant), then uncontrolled dataset heterogeneity, such as technical variation affecting asymmetry measurement, is likely to have been involved. Our observations on the reproducibility of nonsignificant effects highlight the importance of reporting negative findings in publications.

We used ICC of regional thickness and surface area measures from 423 twice‐scanned participants to understand whether inter‐regional differences in imaging quality might relate to inter‐regional differences in reproducibility. There were no significant correlations for either thickness or surface area measures, which is consistent with the relatively high overall reliability of these regional measures. There were also no significant correlations between reproducibility and regional size. These observations again underline that dataset size and true effect size were the main drivers of reproducibility identifiable in the present study. Nonetheless, measurement reliability has previously been shown to play a role in reproducibility, given a specific, true effect size and sample size (Zuo, Xu, & Milham, 2019). A limitation of the present study was the focus on cortical asymmetry effects for cortical gray matter thickness and surface area measures, but other MRI‐based metrics will likely differ in the degree to which measurement reliability affects reproducibility. Therefore we still recommend careful assessment and optimization of measurement reliability in MRI studies.

## 5 CONCLUSION

Reproducing results is critical for accumulating knowledge in the scientific community. In this study, we revisited the outputs of a global collaborative project for mapping cortical brain asymmetry (Kong et al., 2018), to empirically demonstrate reproducibility in a real‐world setting as regards dataset heterogeneity and sample sizes, but in the absence of *p*‐hacking or reporting bias. The results indicated that there is substantial room to improve reproducibility using current neuroimaging methods, even in the absence of *p*‐hacking or reporting bias, because dataset sample size and effect size remained the primary drivers of reproducibility, even in the presence of substantial heterogeneity across datasets. Despite our focus on gray matter asymmetries, this picture is likely to hold true to some extent across the field of brain imaging in general. Further studies will be needed to evaluate reproducibility in more contexts, in terms of imaging modality, processing and measurement techniques, and participant demographics. Our findings suggest that improved reproducibility can be achieved primarily through increasing statistical power, either through increasing the sample sizes of individual datasets, or via collaborations between researchers, for example in consortia such as ENIGMA (Thompson et al., 2014, 2020).

## CONFLICT OF INTERESTS

Disclosures are listed in the Supporting Information Appendix.

## AUTHOR CONTRIBUTIONS

X.‐Z. K. conceived this study and performed data analyses; X.‐Z. K, ELWG, and C. F. contributed data; X. Z. K. and C. F. designed the study, interpreted the results, and wrote the article. All authors provided feedback on the article.

## Supporting information


**Appendix S1** Supporting information

**Figure S1** (See separate pdf file for this figure): Distributions of single‐dataset effect sizes, in relation to sample size. Effect sizes plotted against the left‐hand *y* axis in red, green, and black indicate significant positive, significant negative, and nonsignificant effects in individual datasets (with a significance threshold of *p* < .05). The horizontal dashed line in each plot indicates the effect size from meta‐analysis. The statistical power function (right *y* axis) is superimposed onto each plot based on the meta‐analytic effect size and sample size. In the plot headings, + indicates a significant positive effect in meta‐analysis, − indicates a significant negative effect in meta‐analysis, and o indicates a nonsignificant effect in meta‐analysis.

## Data Availability

Data used in this study were published summary statistics from the ENIGMA cortical asymmetry project (Kong et al., 2018). Data and scripts for all analyses are available in GitHub (https://github.com/Conxz/illusReproducibility). Additional data were from the Human Connectome Project (https://www.humanconnectome.org/), and the BIG dataset (http://cognomics.nl/).

## References

[hbm25154-bib-0001] Aarts, A. A. , Anderson, J. E. , Anderson, C. J. , Attridge, P. R. , Attwood, A. , Axt, J. , … Collaboration, O.S . (2015). Estimating the reproducibility of psychological science. Science, 349(6251), aac4716. 26315443 10.1126/science.aac4716

[hbm25154-bib-0002] Baker, M. (2016). 1,500 scientists lift the lid on reproducibility. Nature, 533, 452–454. 27225100 10.1038/533452a

[hbm25154-bib-0003] Bakker, M. , van Dijk, A. , & Wicherts, J. M. (2012). The rules of the game called psychological science. Perspectives on Psychological Science, 7, 543–554. 26168111 10.1177/1745691612459060

[hbm25154-bib-0004] Benjamin, D. J. , Berger, J. O. , Johannesson, M. , Nosek, B. A. , Wagenmakers, E. J. , Berk, R. , … Johnson, V. E. (2018). Redefine statistical significance. Nature Human Behaviour, 2, 6–10. 10.1038/s41562-017-0189-z 30980045

[hbm25154-bib-0005] Boedhoe, P. S. W. , Heymans, M. W. , Schmaal, L. , Abe, Y. , Alonso, P. , Ameis, S. H. , … Twisk, J. W. R. (2018). An empirical comparison of meta‐ and mega‐analysis with data from the ENIGMA obsessive‐compulsive disorder working Group. Frontiers in Neuroinformatics, 12, 102. 30670959 10.3389/fninf.2018.00102 PMC6331928

[hbm25154-bib-0006] Botvinik‐Nezer, R. , Holzmeister, F. , Camerer, C. F. , Dreber, A. , Huber, J. , Johannesson, M. , … Schonberg, T. (2020). Variability in the analysis of a single neuroimaging dataset by many teams. Nature, 582, 84–88. 32483374 10.1038/s41586-020-2314-9 PMC7771346

[hbm25154-bib-0007] Button, K. S. , Ioannidis, J. P. A. , Mokrysz, C. , Nosek, B. A. , Flint, J. , Robinson, E. S. J. , & Munafo, M. R. (2013). Power failure: Why small sample size undermines the reliability of neuroscience. Nature Reviews Neuroscience, 14, 365–376. 23571845 10.1038/nrn3475

[hbm25154-bib-0008] Carrion‐Castillo, A. , Pepe, A. , Kong, X.‐Z. , Fisher, S. E. , Mazoyer, B. , Tzourio‐Mazoyer, N. , … Francks, C. (2020). Genetic effects on planum temporale asymmetry and their limited relevance to neurodevelopmental disorders, intelligence or educational attainment. Cortex, 124, 137–153. 31887566 10.1016/j.cortex.2019.11.006

[hbm25154-bib-0009] Desmond, J. E. , & Glover, G. H. (2002). Estimating sample size in functional MRI (fMRI) neuroimaging studies: Statistical power analyses. Journal of Neuroscience Methods, 118, 115–128. 12204303 10.1016/s0165-0270(02)00121-8

[hbm25154-bib-0010] Fischl, B. (2012). FreeSurfer. NeuroImage, 62, 774–781. 22248573 10.1016/j.neuroimage.2012.01.021 PMC3685476

[hbm25154-bib-0011] Glasser, M. F. , Sotiropoulos, S. N. , Wilson, J. A. , Coalson, T. S. , Fischl, B. , Andersson, J. L. , … Consortium, W.U.‐M.H . (2013). The minimal preprocessing pipelines for the human connectome project. NeuroImage, 80, 105–124. 23668970 10.1016/j.neuroimage.2013.04.127 PMC3720813

[hbm25154-bib-0012] Gronenschild, E. H. B. M. , Habets, P. , Jacobs, H. I. L. , Mengelers, R. , Rozendaal, N. , van Os, J. , & Marcelis, M. (2012). The effects of FreeSurfer version, workstation type, and Macintosh operating system version on anatomical volume and cortical thickness measurements. PLoS One, 7, e38234. 22675527 10.1371/journal.pone.0038234 PMC3365894

[hbm25154-bib-0013] Ioannidis, J. P. , Munafo, M. R. , Fusar‐Poli, P. , Nosek, B. A. , & David, S. P. (2014). Publication and other reporting biases in cognitive sciences: Detection, prevalence, and prevention. Trends in Cognitive Sciences, 18, 235–241. 24656991 10.1016/j.tics.2014.02.010 PMC4078993

[hbm25154-bib-0014] Ioannidis, J. P. A. (2005). Why most published research findings are false. PLoS Medicine, 2, 696–701. 10.1371/journal.pmed.0020124 PMC1182327 16060722

[hbm25154-bib-0015] Ioannidis, J. P. A. (2008). Why most discovered true associations are inflated. Epidemiology, 19, 640–648. 18633328 10.1097/EDE.0b013e31818131e7

[hbm25154-bib-0016] Iscan, Z. , Jin, T. B. , Kendrick, A. , Szeglin, B. , Lu, H. , Trivedi, M. , … DeLorenzo, C. (2015). Test–retest reliability of freesurfer measurements within and between sites: Effects of visual approval process. Human Brain Mapping, 36, 3472–3485. 26033168 10.1002/hbm.22856 PMC4545736

[hbm25154-bib-0017] John, L. K. , Loewenstein, G. , & Prelec, D. (2012). Measuring the prevalence of questionable research practices with incentives for truth telling. Psychological Science, 23, 524–532. 22508865 10.1177/0956797611430953

[hbm25154-bib-0018] Kharabian Masouleh, S. , Eickhoff, S. B. , Hoffstaedter, F. , Genon, S. , & Alzheimer's Disease Neuroimaging, I . (2019). Empirical examination of the replicability of associations between brain structure and psychological variables. eLife, 8, e43464. 30864950 10.7554/eLife.43464 PMC6483597

[hbm25154-bib-0019] Klein, O. , Hardwicke, T. E. , Aust, F. , Breuer, J. , Danielsson, H. , Mohr, A. H. , … Frank, M. C. (2018). A practical guide for transparency in psychological science. Collabra: Psychology, 4(1), 20.

[hbm25154-bib-0020] Klein, R. , Vianello, M. , Hasselman, F. , Adams, B. , Adams, R.B. , Alper, S. , …, Nosek, B. (2018). Many labs 2: Investigating variation in replicability across sample and setting. *PsyArXiv*.

[hbm25154-bib-0021] Klein, R. A. , Ratliff, K. A. , Vianello, M. , Adams, R. B. , Bahnik, S. , Bernstein, M. J. , … Nosek, B. A. (2014). Investigating variation in replicability a “many labs” replication project. Social Psychology—Germany, 45, 142–152.

[hbm25154-bib-0022] Kong, X. Z. , Boedhoe, P. S. W. , Abe, Y. , Alonso, P. , Ameis, S. H. , Arnold, P. D. , … Francks, C. (2020). Mapping cortical and subcortical asymmetry in obsessive‐compulsive disorder: Findings from the ENIGMA Consortium. Biological Psychiatry, 87(12), 1022–1024. 31178097 10.1016/j.biopsych.2019.04.022 PMC7094802

[hbm25154-bib-0023] Kong, X. Z. , Mathias, S. R. , Guadalupe, T. , Group, E. L. W. , Glahn, D. C. , Franke, B. , … Francks, C. (2018). Mapping cortical brain asymmetry in 17,141 healthy individuals worldwide via the ENIGMA Consortium. Proceedings of the National Academy of Sciences of the United States of America, 115, E5154–E5163. 29764998 10.1073/pnas.1718418115 PMC5984496

[hbm25154-bib-0024] Kong, X.‐Z. , Postema, M. , Guadalupe, T. , Kovel, C.d. , Boedhoe, P.S. , Hoogman, M. , …, Francks, C. (2019). Mapping brain asymmetry in health and disease through the ENIGMA consortium. *Human Brain Mapping*, 1–15. 10.1002/hbm.25033. PMC8675409 32420672

[hbm25154-bib-0026] LeWinn, K. Z. , Sheridan, M. A. , Keyes, K. M. , Hamilton, A. , & McLaughlin, K. A. (2017). Sample composition alters associations between age and brain structure. Nature Communications, 8, 874. 10.1038/s41467-017-00908-7 PMC5638928 29026076

[hbm25154-bib-0025] Mapping brain asymmetry in health and disease through the ENIGMA consortium. Human Brain Mapping, 2020, 1–15. 10.1002/hbm.25033. PMC8675409 32420672

[hbm25154-bib-0027] Nosek, B. A. , Alter, G. , Banks, G. C. , Borsboom, D. , Bowman, S. D. , Breckler, S. J. , … Yarkoni, T. (2015). Promoting an open research culture. Science, 348, 1422–1425. 26113702 10.1126/science.aab2374 PMC4550299

[hbm25154-bib-0028] Pauli, R. , Bowring, A. , Reynolds, R. , Chen, G. , Nichols, T. E. , & Maumet, C. (2016). Exploring fMRI results space: 31 variants of an fMRI analysis in AFNI, FSL, and SPM. Frontiers in Neuroinformatics, 10, 1–6. 27458367 10.3389/fninf.2016.00024 PMC4932120

[hbm25154-bib-0029] Poldrack, R. A. , Baker, C. I. , Durnez, J. , Gorgolewski, K. J. , Matthews, P. M. , Munafo, M. R. , … Yarkoni, T. (2017). Scanning the horizon: Towards transparent and reproducible neuroimaging research. Nature Reviews Neuroscience, 18, 115–126. 28053326 10.1038/nrn.2016.167 PMC6910649

[hbm25154-bib-0030] Postema, M. C. , van Rooij, D. , Anagnostou, E. , Arango, C. , Auzias, G. , Behrmann, M. , … Francks, C. (2019). Altered structural brain asymmetry in autism spectrum disorder in a study of 54 datasets. Nature Communications, 10, 4958. 10.1038/s41467-019-13005-8 PMC6823355 31673008

[hbm25154-bib-0031] Prinz, F. , Schlange, T. , & Asadullah, K. (2011). Believe it or not: How much can we rely on published data on potential drug targets? Nature Reviews. Drug Discovery, 10, 712–U81. 10.1038/nrd3439-c1 21892149

[hbm25154-bib-0032] Simmons, J. P. , Nelson, L. D. , & Simonsohn, U. (2011). False‐positive psychology: Undisclosed flexibility in data collection and analysis allows presenting anything as significant. Psychological Science, 22, 1359–1366. 22006061 10.1177/0956797611417632

[hbm25154-bib-0033] Thompson, P. M. , Jahanshad, N. , Ching, C. R. K. , Salminen, L. E. , Thomopoulos, S. I. , Bright, J. , … Consortium, E. (2020). ENIGMA and global neuroscience: A decade of large‐scale studies of the brain in health and disease across more than 40 countries. Translational Psychiatry, 10, 100. 32198361 10.1038/s41398-020-0705-1 PMC7083923

[hbm25154-bib-0034] Thompson, P. M. , Stein, J. L. , Medland, S. E. , Hibar, D. P. , Vasquez, A. A. , Renteria, M. E. , … Alzheimer's Disease Neuroimaging Initiative, E.C.I.C.S.Y.S.G . (2014). The ENIGMA Consortium: Large‐scale collaborative analyses of neuroimaging and genetic data. Brain Imaging and Behavior, 8, 153–182. 24399358 10.1007/s11682-013-9269-5 PMC4008818

[hbm25154-bib-0035] Toga, A. W. , & Thompson, P. M. (2003). Mapping brain asymmetry. Nature Reviews. Neuroscience, 4, 37–48. 12511860 10.1038/nrn1009

[hbm25154-bib-0036] Valentin Amrhein, S. G. (2017). Remove, rather than redefine, statistical significance. Nature Human Behaviour, 2, 4. 10.1038/s41562-017-0224-0 30980046

[hbm25154-bib-0037] Wager, T. D. , Lindquist, M. A. , Nichols, T. E. , Kober, H. , & Van Snellenberg, J. X. (2009). Evaluating the consistency and specificity of neuroimaging data using meta‐analysis. NeuroImage, 45, S210–S221. 19063980 10.1016/j.neuroimage.2008.10.061 PMC3318962

[hbm25154-bib-0038] Zhen, Z. , Kong, X. Z. , Huang, L. , Yang, Z. , Wang, X. , Hao, X. , … Liu, J. (2017). Quantifying the variability of scene‐selective regions: Interindividual, interhemispheric, and sex differences. Human Brain Mapping, 38, 2260–2275. 28117508 10.1002/hbm.23519 PMC6866930

[hbm25154-bib-0039] Zuo, X. N. , Xu, T. , & Milham, M. P. (2019). Harnessing reliability for neuroscience research. Nature Human Behaviour, 3, 768–771. 10.1038/s41562-019-0655-x 31253883

