# Supplementary material for: Reproducibility in the absence of selective reporting: An illustration from large‐scale brain asymmetry research
Source: Hum Brain Mapp. 2020 Aug 25;43(1):244–54. doi: 10.1002/hbm.25154 (PMC8675427; doi:10.1002/hbm.25154)
Supplement: Supplementary file 2 — Figure S1 (See separate pdf file for this figure): Distributions of single‐dataset effect sizes, in relation to sample size. Effect sizes plotted against the left‐hand y axis in red, green, and black indicate significant positive, significant negative, and nonsignificant effects in individual datasets (with a significance threshold of p < .05). The horizontal dashed line in each plot indicates the effect size from meta‐analysis. The statistical power function (right y axis) is superimposed onto each plot based on the meta‐analytic effect size and sample size. In the plot headings, + indicates a significant positive effect in meta‐analysis, − indicates a significant negative effect in meta‐analysis, and o indicates a nonsignificant effect in meta‐analysis. [file HBM-43-244-s001.pdf]

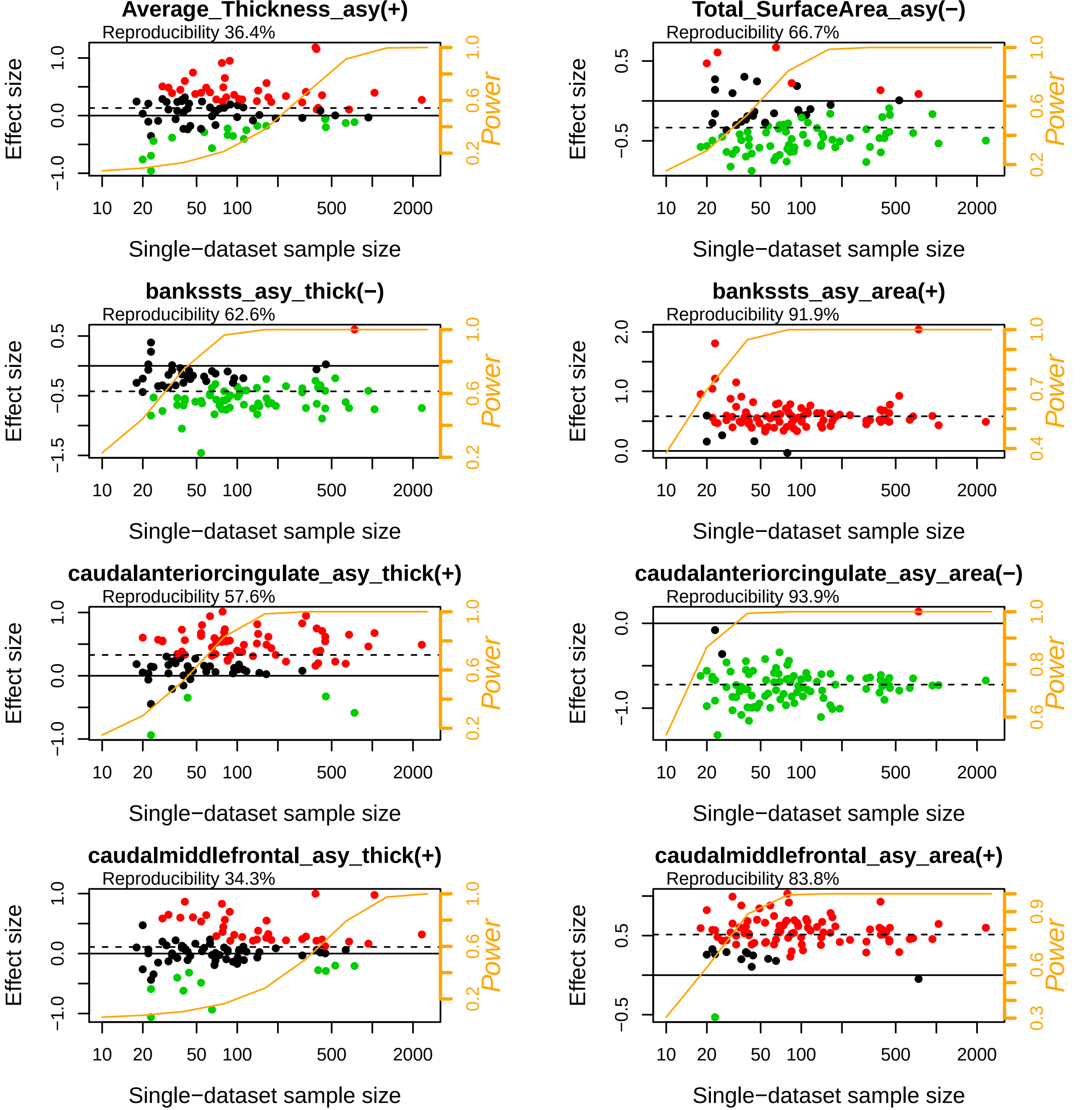
Average_Thickness_asy(+)
Reproducibility 36.4%
Total_SurfaceArea_asy(−)
Reproducibility 66.7%
bankssts_asy_thick(−)
Reproducibility 62.6%
bankssts_asy_area(+)
Reproducibility 91.9%
caudalanteriorcingulate_asy_thick(+)
Reproducibility 57.6%
caudalanteriorcingulate_asy_area(−)
Reproducibility 93.9%
caudalmiddlefrontal_asy_thick(+)
Reproducibility 34.3%
caudalmiddlefrontal_asy_area(+)
Reproducibility 83.8%
Effect size
Power
Single−dataset sample size
10
20
50
100
500
2000

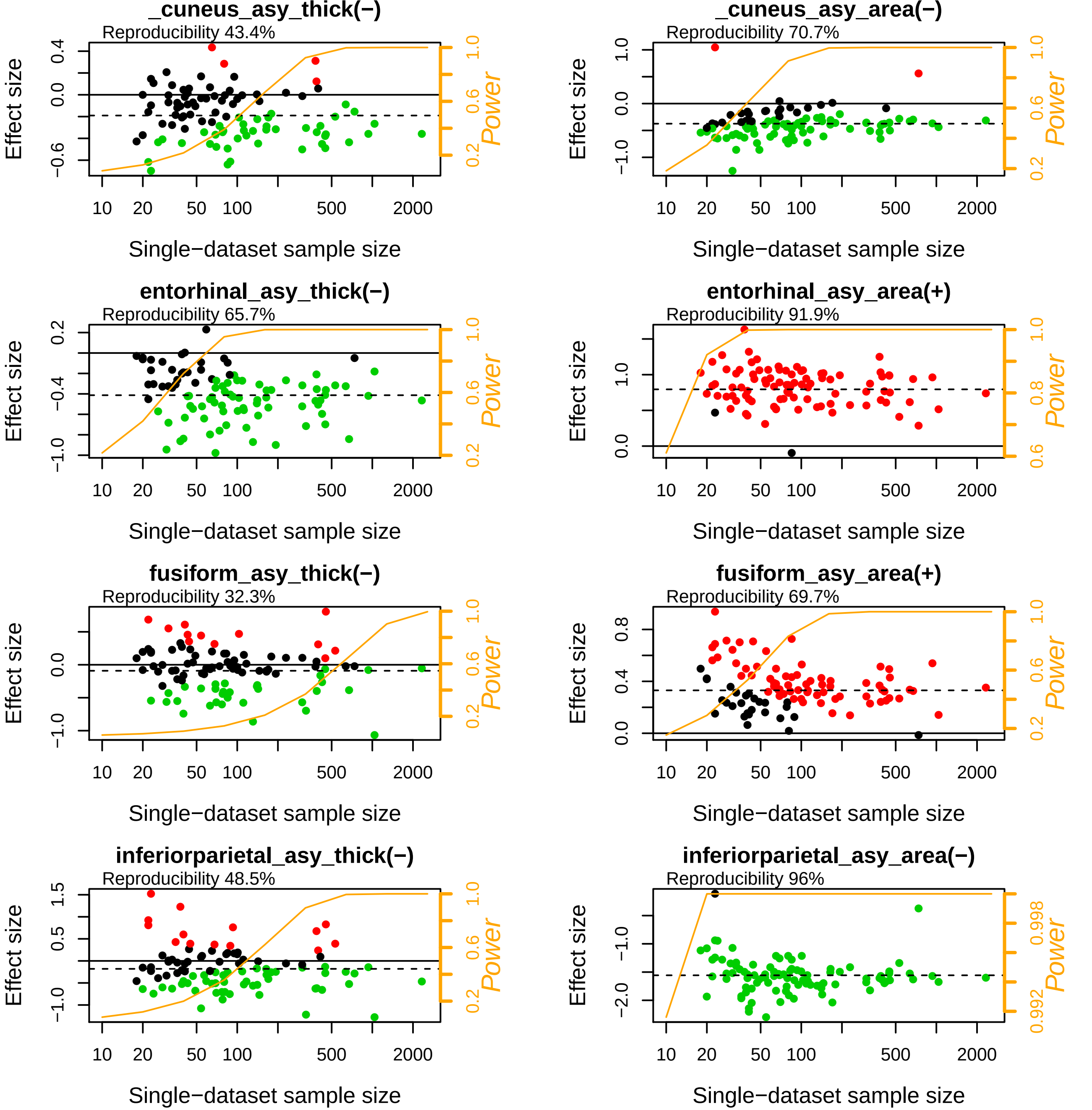

_cuneus_asy_thick(−)
Reproducibility 43.4%
_cuneus_asy_area(−)
Reproducibility 70.7%
entorhinal_asy_thick(−)
Reproducibility 65.7%
entorhinal_asy_area(+)
Reproducibility 91.9%
fusiform_asy_thick(−)
Reproducibility 32.3%
fusiform_asy_area(+)
Reproducibility 69.7%
inferiorparietal_asy_thick(−)
Reproducibility 48.5%
inferiorparietal_asy_area(−)
Reproducibility 96%
Effect size
Power
Single−dataset sample size
10
20
50
100
500
2000

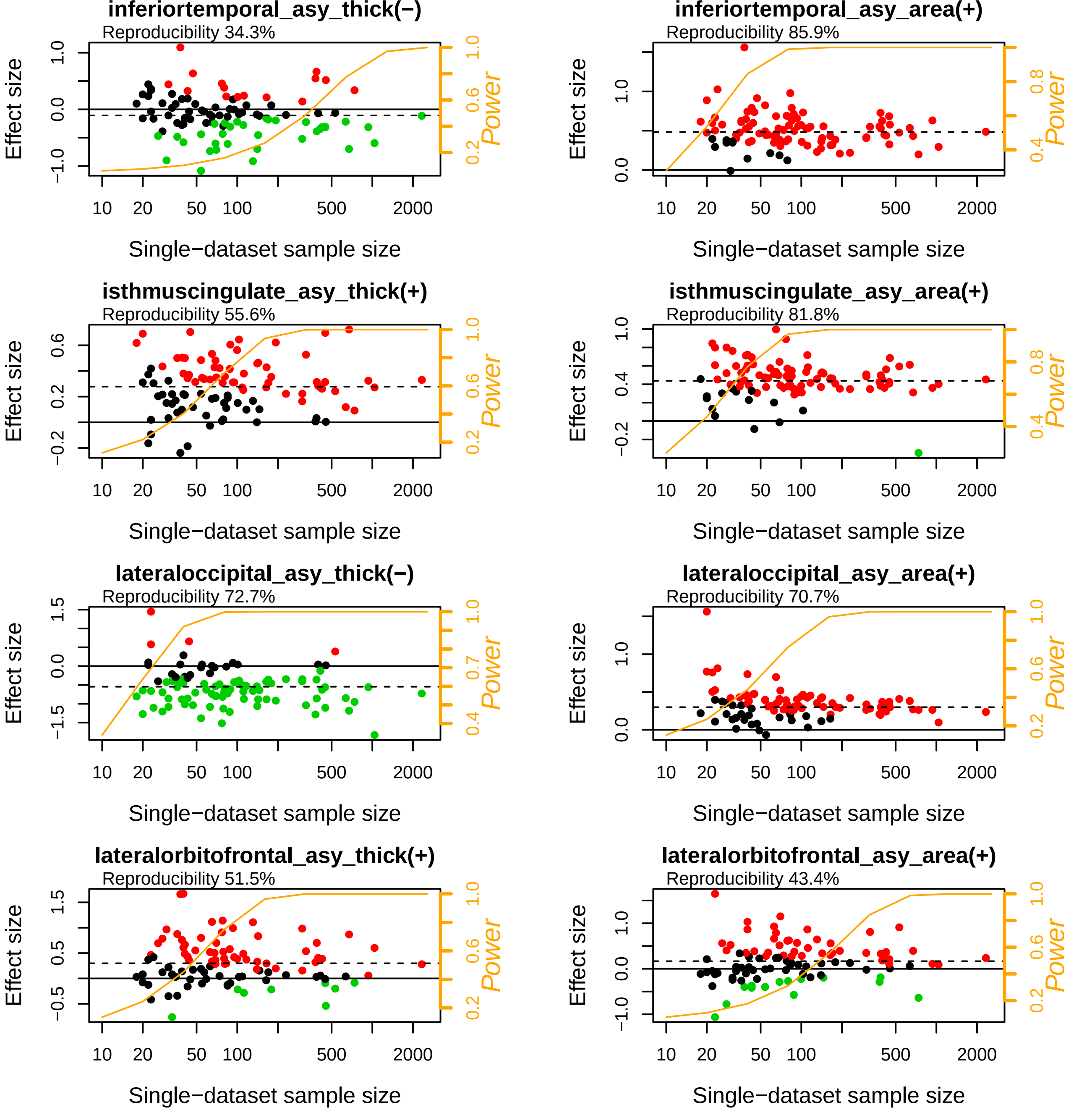

inferiortemporal_asy_thick(−)
Reproducibility 34.3%
inferiortemporal_asy_area(+)
Reproducibility 85.9%
isthmuscingulate_asy_thick(+)
Reproducibility 55.6%
isthmuscingulate_asy_area(+)
Reproducibility 81.8%
lateraloccipital_asy_thick(−)
Reproducibility 72.7%
lateraloccipital_asy_area(+)
Reproducibility 70.7%
lateralorbitofrontal_asy_thick(+)
Reproducibility 51.5%
lateralorbitofrontal_asy_area(+)
Reproducibility 43.4%
Effect size
Power
Single−dataset sample size
10
20
50
100
500
2000

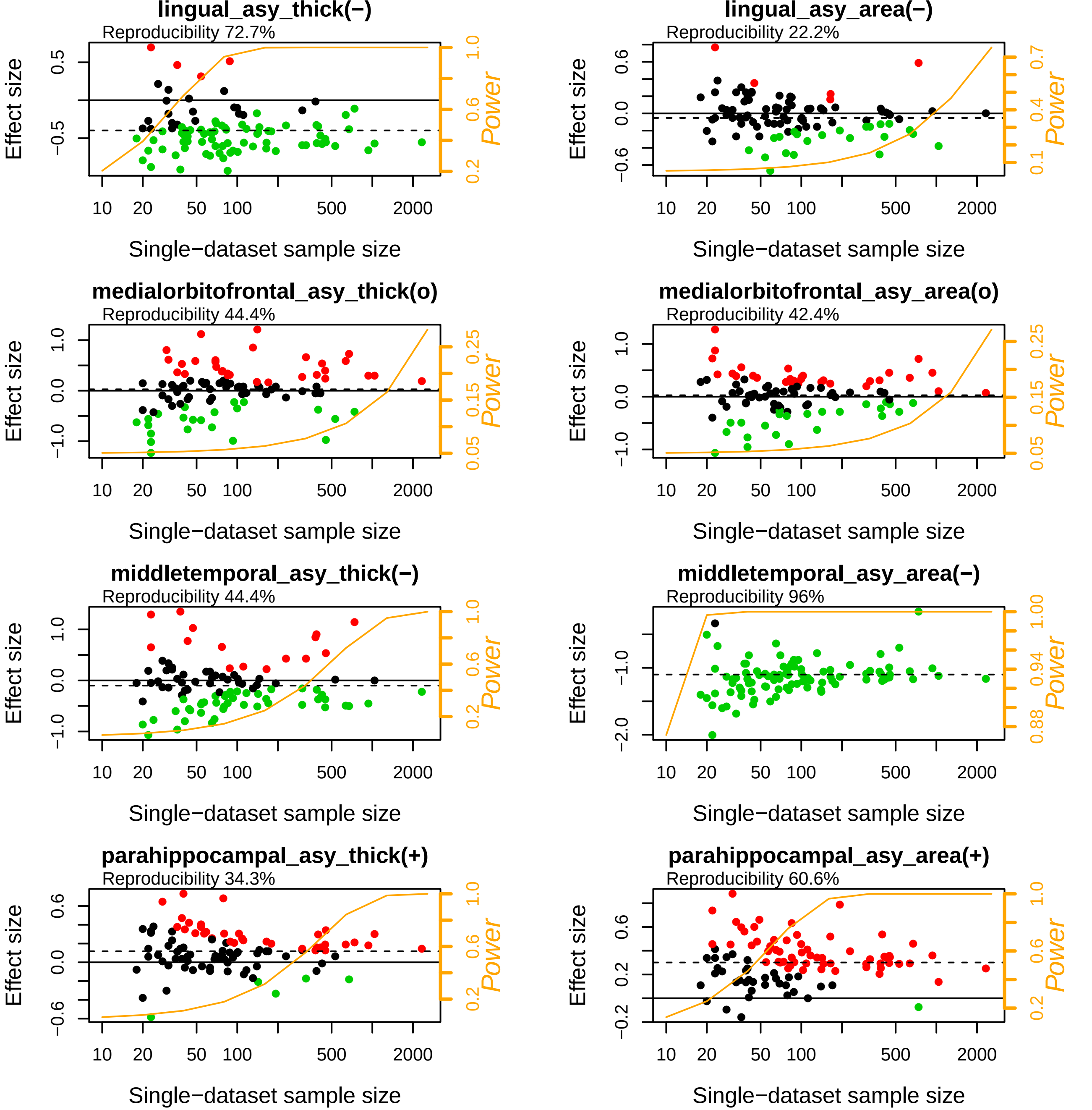
lingual_asy_thick(−)
Reproducibility 72.7%
lingual_asy_area(−)
Reproducibility 22.2%
medialorbitofrontal_asy_thick(o)
Reproducibility 44.4%
medialorbitofrontal_asy_area(o)
Reproducibility 42.4%
middletemporal_asy_thick(−)
Reproducibility 44.4%
middletemporal_asy_area(−)
Reproducibility 96%
parahippocampal_asy_thick(+)
Reproducibility 34.3%
parahippocampal_asy_area(+)
Reproducibility 60.6%
Effect size
Power
Single−dataset sample size
10 20 50 100 500 2000

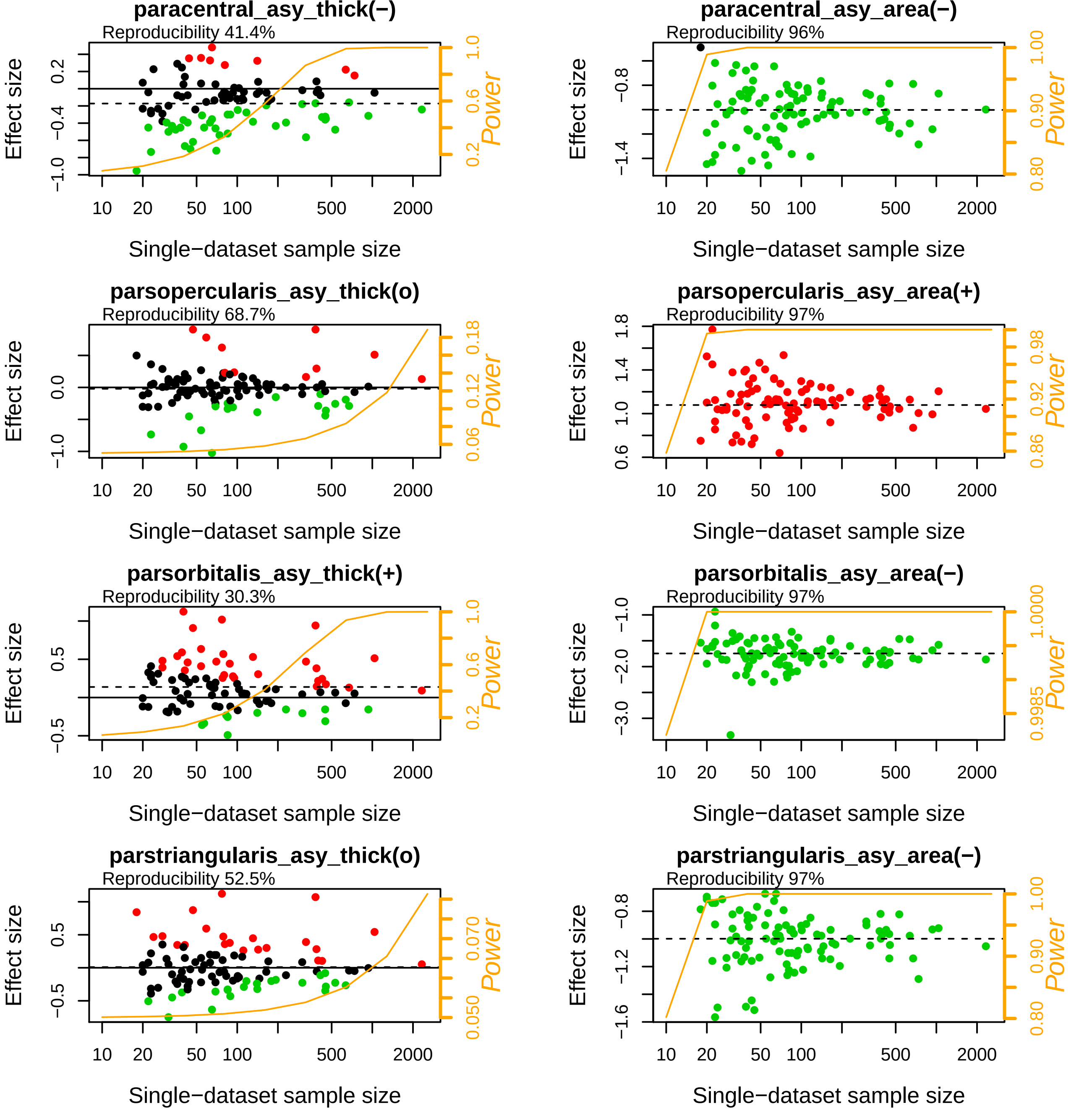
paracentral_asy_thick(−)
Reproducibility 41.4%
paracentral_asy_area(−)
Reproducibility 96%
parsopercularis_asy_thick(o)
Reproducibility 68.7%
parsopercularis_asy_area(+)
Reproducibility 97%
parsorbitalis_asy_thick(+)
Reproducibility 30.3%
parsorbitalis_asy_area(−)
Reproducibility 97%
parstriangularis_asy_thick(o)
Reproducibility 52.5%
parstriangularis_asy_area(−)
Reproducibility 97%
Effect size
Power
Single−dataset sample size
10
20
50
100
500
2000

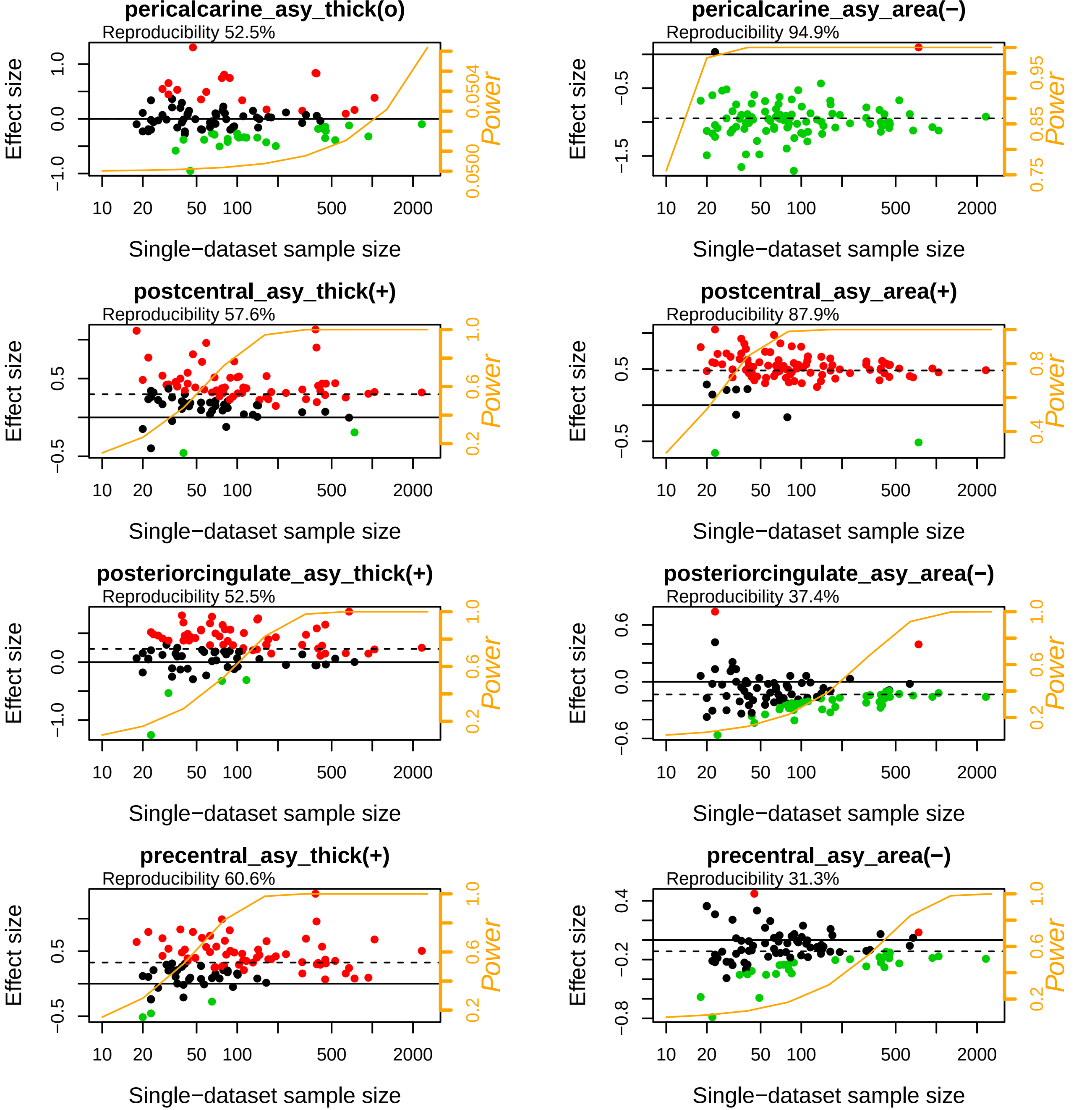
pericalcarine_asy_thick(o)
Reproducibility 52.5%
pericalcarine_asy_area(−)
Reproducibility 94.9%
postcentral_asy_thick(+)
Reproducibility 57.6%
postcentral_asy_area(+)
Reproducibility 87.9%
posteriorcingulate_asy_thick(+)
Reproducibility 52.5%
posteriorcingulate_asy_area(−)
Reproducibility 37.4%
precentral_asy_thick(+)
Reproducibility 60.6%
precentral_asy_area(−)
Reproducibility 31.3%
Effect size
Power
Single−dataset sample size
10 20 50 100 500 2000

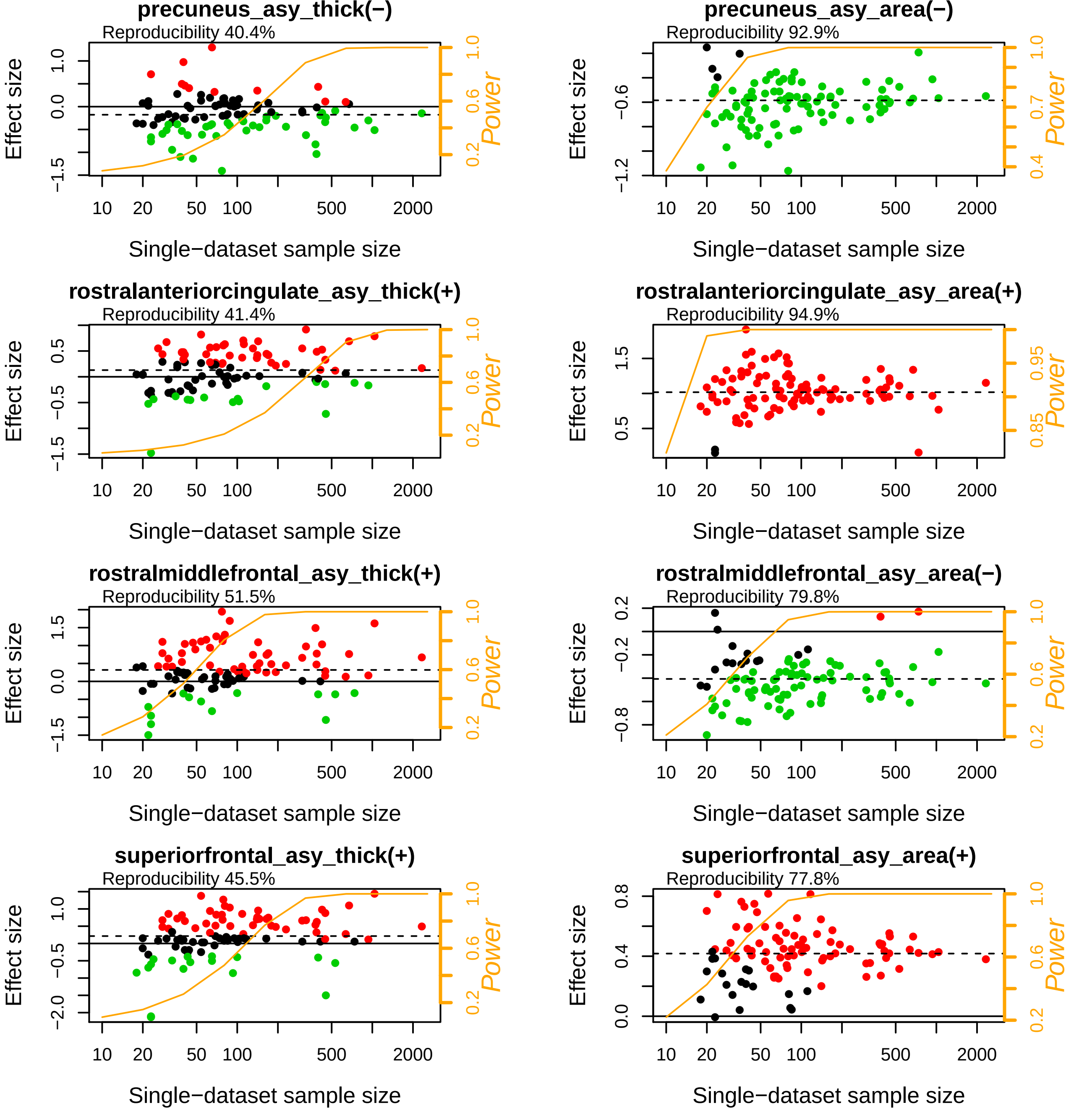
precuneus_asy_thick(−)
Reproducibility 40.4%
precuneus_asy_area(−)
Reproducibility 92.9%
rostralanteriorcingulate_asy_thick(+)
Reproducibility 41.4%
rostralanteriorcingulate_asy_area(+)
Reproducibility 94.9%
rostralmiddlefrontal_asy_thick(+)
Reproducibility 51.5%
rostralmiddlefrontal_asy_area(−)
Reproducibility 79.8%
superiorfrontal_asy_thick(+)
Reproducibility 45.5%
superiorfrontal_asy_area(+)
Reproducibility 77.8%
Effect size
Power
Single−dataset sample size
10
20
50
100
500
2000

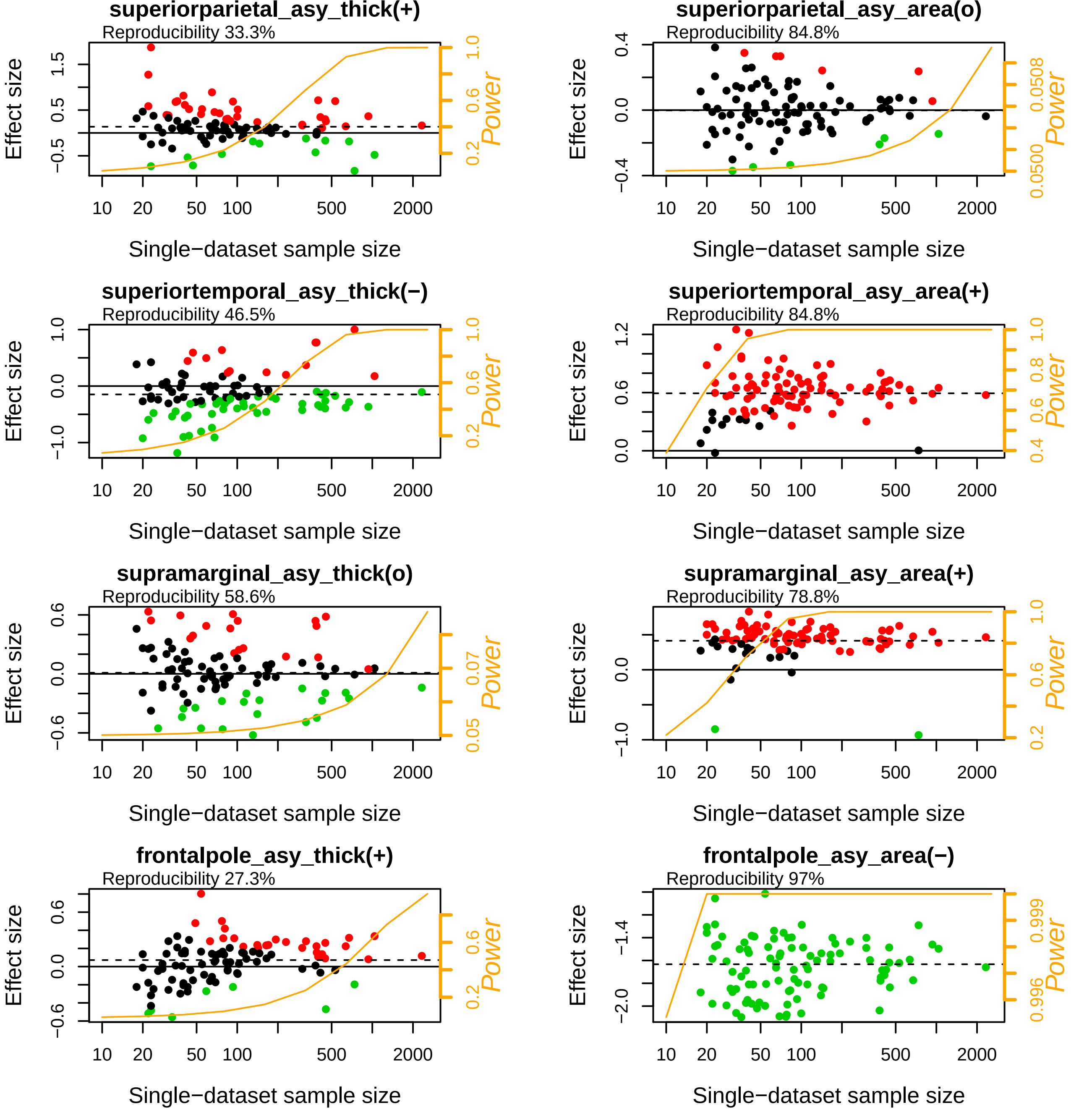

superiorparietal_asy_thick(+)
Reproducibility 33.3%
Effect size
1.5
0.5
−0.5
Power
0.2
0.6
1.0
10
20
50
100
500
2000
Single−dataset sample size
superiorparietal_asy_area(o)
Reproducibility 84.8%
Effect size
0.4
0.0
−0.4
Power
0.0500
0.0508
Single−dataset sample size
superiortemporal_asy_thick(−)
Reproducibility 46.5%
Effect size
1.0
0.0
−1.0
Power
Single−dataset sample size
superiortemporal_asy_area(+)
Reproducibility 84.8%
Effect size
1.2
0.6
0.0
Power
0.4
0.6
0.8
1.0
Single−dataset sample size
supramarginal_asy_thick(o)
Reproducibility 58.6%
Effect size
0.6
0.0
−0.6
Power
0.05
0.07
Single−dataset sample size
supramarginal_asy_area(+)
Reproducibility 78.8%
Effect size
0.0
−1.0
Power
0.2
0.6
1.0
Single−dataset sample size
frontalpole_asy_thick(+)
Reproducibility 27.3%
Effect size
0.6
0.0
−0.6
Power
0.2
0.6
Single−dataset sample size
frontalpole_asy_area(−)
Reproducibility 97%
Effect size
−1.4
−2.0
Power
0.996
0.999
Single−dataset sample size

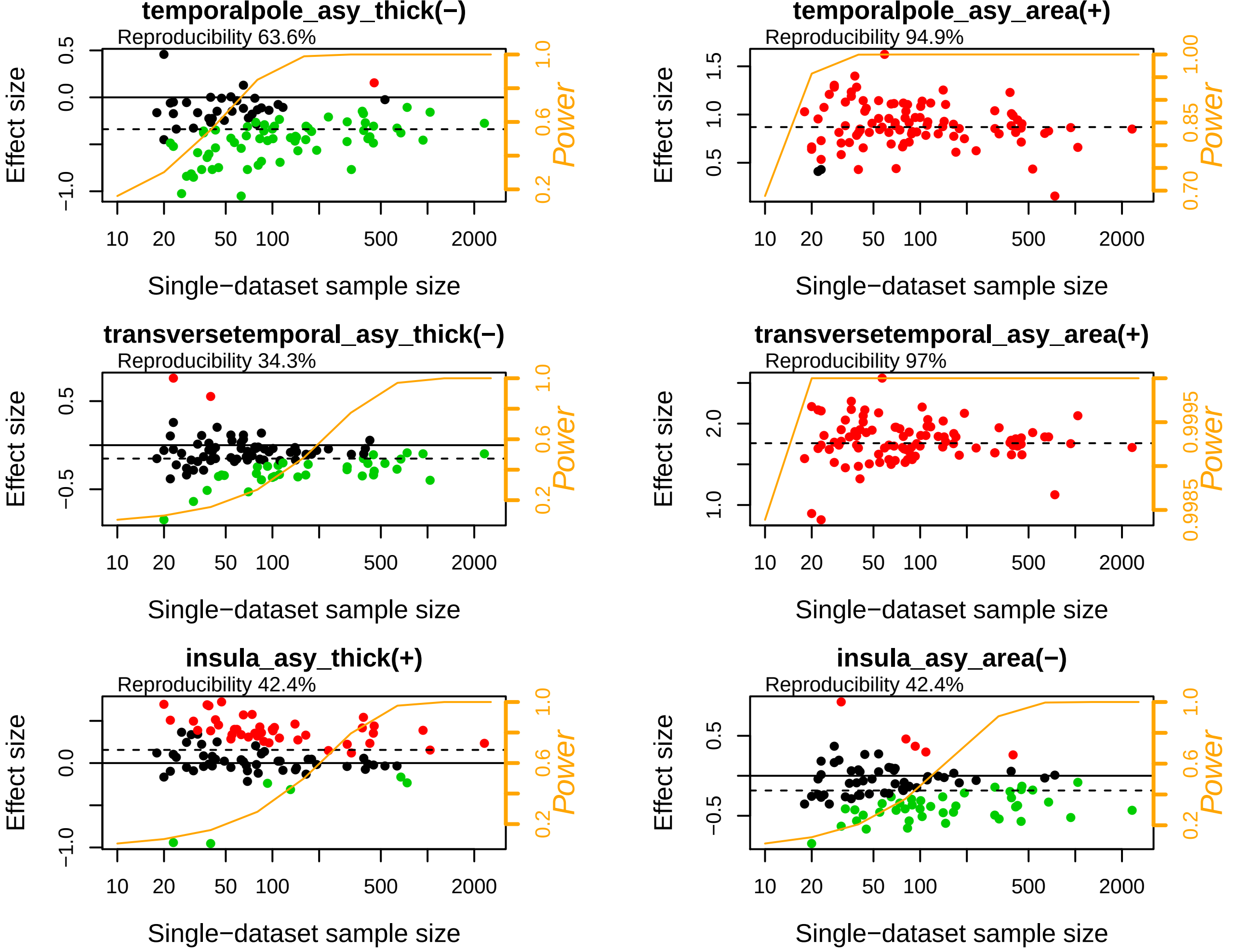

temporalpole_asy_thick(−)
Reproducibility 63.6%
temporalpole_asy_area(+)
Reproducibility 94.9%
transversetemporal_asy_thick(−)
Reproducibility 34.3%
transversetemporal_asy_area(+)
Reproducibility 97%
insula_asy_thick(+)
Reproducibility 42.4%
insula_asy_area(−)
Reproducibility 42.4%
Effect size
Power
Single−dataset sample size
10
20
50
100
500
2000
